# Minocycline at 2 Different Dosages vs Placebo for Patients With Mild Alzheimer Disease: A Randomized Clinical Trial

**DOI:** 10.1001/jamaneurol.2019.3762

**Published:** 2019-11-18

**Authors:** Robert Howard, Olga Zubko, Rosie Bradley, Emma Harper, Lynn Pank, John O’Brien, Chris Fox, Naji Tabet, Gill Livingston, Peter Bentham, Rupert McShane, Alistair Burns, Craig Ritchie, Suzanne Reeves, Simon Lovestone, Clive Ballard, Wendy Noble, Ramin Nilforooshan, Gordon Wilcock, Richard Gray

**Affiliations:** 1 Division of Psychiatry, University College London, London, United Kingdom; 2 Old Age Psychiatry, King’s College London, London, United Kingdom; 3 Medical Research Council Population Health Research Unit, University of Oxford, Oxford, United Kingdom; 4 Nuffield Department of Population Health, University of Oxford, Oxford, United Kingdom; 5 Department of Psychiatry, University of Cambridge, Cambridge, United Kingdom; 6 Norwich Medical School, University of East Anglia, Norwich, United Kingdom; 7 Department of Old Age Psychiatry, University of Sussex, Brighton, United Kingdom; 8 Division of Psychiatry, University College London, London, United Kingdom; 9 Birmingham and Solihull Mental Health National Health Service Foundation Trust, Birmingham, United Kingdom; 10 Department of Psychiatry, University of Oxford, Oxford, United Kingdom; 11 Department of Old Age Psychiatry, University of Manchester, Manchester, United Kingdom; 12 Centre for Clinical Brain Sciences, University of Edinburgh, Edinburgh, United Kingdom; 13 Medical School, University of Exeter, Exeter, United Kingdom; 14 Department of Basic and Clinical Neuroscience, King’s College London, London, United Kingdom; 15 Surrey and Borders Partnership National Health Service Foundation Trust, United Kingdom; 16 Department of Clinical Neurosciences, University of Oxford, Oxford, United Kingdom

## Abstract

**Importance:**

There are no disease-modifying treatments for Alzheimer disease (AD), the most common cause of dementia. Minocycline is anti-inflammatory, protects against the toxic effects of β-amyloid in vitro and in animal models of AD, and is a credible repurposed treatment candidate.

**Objective:**

To determine whether 24 months of minocycline treatment can modify cognitive and functional decline in patients with mild AD.

**Design, Setting, and Participants:**

Participants were recruited into a double-blind randomized clinical trial from May 23, 2014, to April 14, 2016, with 24 months of treatment and follow-up. This multicenter study in England and Scotland involved 32 National Health Service memory clinics within secondary specialist services for people with dementia. From 886 screened patients, 554 patients with a diagnosis of mild AD (Standardised Mini-Mental State Examination [sMMSE] score ≥24) were randomized.

**Interventions:**

Participants were randomly allocated 1:1:1 in a semifactorial design to receive minocycline (400 mg/d or 200 mg/d) or placebo for 24 months.

**Main Outcomes and Measures:**

Primary outcome measures were decrease in sMMSE score and Bristol Activities of Daily Living Scale (BADLS), analyzed by intention-to-treat repeated-measures regression.

**Results:**

Of 544 eligible participants (241 women and 303 men), the mean (SD) age was 74.3 (8.2) years, and the mean (SD) sMMSE score was 26.4 (1.9). Fewer participants completed 400-mg minocycline hydrochloride treatment (28.8% [53 of 184]) than 200-mg minocycline treatment (61.9% [112 of 181]) or placebo (63.7% [114 of 179]; *P* < .001), mainly because of gastrointestinal symptoms (42 in the 400-mg group, 15 in the 200-mg group, and 10 in the placebo group; *P* < .001), dermatologic adverse effects (10 in the 400-mg group, 5 in the 200-mg group, and 1 in the placebo group; *P* = .02), and dizziness (14 in the 400-mg group, 3 in the 200-mg group, and 1 in the placebo group; *P* = .01). Assessment rates were lower in the 400-mg group: 68.4% (119 of 174 expected) for sMMSE at 24 months compared with 81.8% (144 of 176) for the 200-mg group and 83.8% (140 of 167) for the placebo group. Decrease in sMMSE scores over 24 months in the combined minocycline group was similar to that in the placebo group (4.1 vs 4.3 points). The combined minocycline group had mean sMMSE scores 0.1 points higher than the placebo group (95% CI, −1.1 to 1.2; *P* = .90). The decrease in mean sMMSE scores was less in the 400-mg group than in the 200-mg group (3.3 vs 4.7 points; treatment effect = 1.2; 95% CI, −0.1 to 2.5; *P* = .08). Worsening of BADLS scores over 24 months was similar in all groups: 5.7 in the 400-mg group, 6.6 in the 200-mg group, and 6.2 in the placebo groups (treatment effect for minocycline vs placebo = –0.53; 95% CI, −2.4 to 1.3; *P* = .57; treatment effect for 400 mg vs 200 mg of minocycline = –0.31; 95% CI, −0.2 to 1.8; *P* = .77). Results were similar in different patient subgroups and in sensitivity analyses adjusting for missing data.

**Conclusions and Relevance:**

Minocycline did not delay the progress of cognitive or functional impairment in people with mild AD during a 2-year period. This study also found that 400 mg of minocycline is poorly tolerated in this population.

**Trial Registration:**

isrctn.org Identifier: ISRCTN16105064

## Key Points

**Question:**

Can 2 years of minocycline treatment modify the course of mild Alzheimer disease?

**Findings:**

In this randomized clinical trial that included 544 participants, 24 months of minocycline treatment did not significantly delay progression of functional and cognitive impairment compared with placebo.

**Meaning:**

Minocycline is not a candidate for disease modification for patients with symptomatic Alzheimer disease.

## Teaser

This randomized clinical trial examines whether 24 months of minocycline treatment can modify cognitive and functional decline in patients with mild Alzheimer disease.

## Introduction

Alzheimer disease (AD) affects 50 million people worldwide,^1^ with numbers projected to reach 135.5 million by 2050; associated costs for the United States are $1.2 trillion.^2^ At the 2013 Dementia Summit, G8 ministers committed to identifying a cure or disease-modifying therapy by 2025,^2^ but no therapy has so far been shown to delay the progression of cognitive and functional disability. Failure of treatment approaches directed at preventing buildup of β-amyloid (Aβ) or tau has stimulated investigation of alternative treatment approaches, including targeting inflammation.

Alzheimer disease is associated with immune-related and inflammatory genes, including myeloid-specific sialic acid binding receptor (*CD33*), triggering receptor expressed on myeloid cell 2 (*TREM2*), complement receptor 1 (*CR1*), and bridging integrator 1 (*BIN1*).^3^ Microglial activation is increased in AD.^4^ Aβ is a proinflammatory agent in AD,^5^ and microglial surface receptors are also Aβ receptors.^6^ In early AD, microglia clear Aβ by phagocytosis and produce Aβ-degrading enzymes.^7^ However, as AD progresses, accumulation of Aβ stimulates microglial production of proinflammatory agents that are associated with neurodegeneration.^7^

Two systematic reviews, based on expert opinion and tolerability, brain penetration, and preclinical and early phase trial data on repositioned drugs identified minocycline hydrochloride among the high-priority drugs to progress to clinical trials in AD.^8,9^ Minocycline is an anti-inflammatory tetracycline that crosses the blood-brain barrier and inhibits proinflammatory microglia. In vitro, minocycline protects against Aβ-induced cell death and prevents fibrillization of Aβ.^10^ In transgenic mice, minocycline prevents Aβ deposition and neuronal death^11^; reduces tau phosphorylation and insoluble tau aggregates^12^; downregulates inducible nitric oxide synthetase, cyclooxygenase-2, and Aβ precursor protein cleaving enzyme-1^13^; and protects hippocampal neurogenesis in the presence of Aβ.^14^ Minocycline reduces interleukin and tumor necrosis factor levels in mice^15^ and neuronal death and learning deficits in rats after Aβ administration.^16^

We investigated whether minocycline slows the decline in cognitive and functional ability in people with mild AD over a 2-year treatment period and whether giving minocycline hydrochloride at a higher (400-mg) dose than the 200 mg used in standard practice enhanced efficacy.

## Methods

### Study Design

The Minocycline in Alzheimer Disease Efficacy (MADE) trial is a double-blind randomized clinical trial of individuals with mild AD that is investigating whether 200 mg or 400 mg of minocycline hydrochloride per day slows the rate of decline in cognitive and functional ability over 2 years compared with placebo. Participants were enrolled from National Health Service memory services from May 23, 2014, to April 14, 2016. Eligible participants had a diagnosis of possible or probable AD,^17^ were older than 50 years, could give informed consent for involvement, had a Standardised Mini-Mental State Examination (sMMSE)^18^ score of 24 to 30, and had a caregiver to supervise medication and complete Bristol Activities of Daily Living Scale (BADLS) assessments.^19^ Exclusions included tetracycline allergy, women of childbearing age, uncontrolled serious concomitant illness, stage 3b to 5 chronic kidney disease, moderate liver disease, systemic lupus erythematosus, and participation in another clinical trial in the previous 28 days. The MADE trial was conducted in accordance with the International Conference on Harmonization Good Clinical Practice guidelines and the Declaration of Helsinki.^20^ Patients provided written informed consent. The study protocol, patient and caregiver information sheets, and informed consent forms were approved by East of England/Essex Research Ethics Committee and the Medicines and Healthcare Products Regulatory Agency (trial protocol in Supplement 1). This study followed the Consolidated Standards of Reporting Trials (CONSORT) reporting guideline.

### Randomization and Masking

Participants were centrally randomized to receive minocycline hydrochloride, 400 mg; minocycline hydrochloride, 200 mg; or placebo. The minimized randomization procedure aimed to balance treatment allocation overall and by 4 stratification variables: center, duration of symptoms prior to randomization (<6 months or ≥6 months), sMMSE score (24-26 or 27-30), and age (<65 years, 65-74 years, or ≥75 years). Participants were enrolled by their clinicians, or appropriately trained clinical study officers, who also administered outcome assessments.

### Trial Procedures

Modified-release capsules, containing 100 mg of minocycline hydrochloride, and identically appearing placebo capsules (Modepharma) in foil blister packs, dispensed every 13 weeks, were used. Trial group dosing was as follows: (1) minocycline hydrochloride, 400 mg (two 100-mg capsules in the morning and the evening); (2) minocycline hydrochloride, 200 mg (one 100-mg capsule plus 1 placebo capsule in the morning and the evening); and (3) placebo (2 placebo capsules in the morning and the evening). Participants, carergivers, prescribing clinicians, outcome assessors, and all trial staff members (except statisticians) were masked to group assignment.

Participants visited the clinic at baseline, week 2, and months 3, 6, 9, 12, 15, 18, 21, and 24. Information on adherence was collected at each assessment and through dispensing records. Adverse events were recorded at each visit. Outcome assessments were at baseline and months 6, 12, 18, and 24.

### Outcome Measures

Co-primary outcomes were rate of decline from baseline to 24 months on sMMSE (scores range from 0 to 30, with higher scores indicating better cognitive function)^21^ and BADLS (scores range from 0 to 60, with higher scores indicating greater impairment).^22^ Secondary outcomes were safety and concurrent infections.

### Statistical Analysis

Predefined primary analyses were of minocycline (any dose) vs placebo and of minocycline hydrochloride 400 mg vs 200 mg. Based on previous studies, we estimated that 24-month assessments would be available for at least 80% of surviving participants (approximately 390 participants), which would provide 90% power at *P* < .05 to detect a small to moderate (0.35 SD) effect size for minocycline (any dose) compared with placebo on the primary outcome measures. With outcome assessments on 130 patients allocated minocycline hydrochloride, 400 mg, and 130 allocated minocycline hydrochloride, 200 mg, we would have 80% power at *P* < .05 to detect a 0.35-SD treatment effect of 400 mg compared with 200 mg at 24 months.

Only participants who received at least 1 capsule of study treatment were to be included in the analyses of primary and secondary outcomes. The primary analyses of the effect of minocycline on the rate of decline of sMMSE and BADLS scores and subgroup analyses used intention-to-treat repeated-measures regression methods, adjusted for baseline scores. These analyses use all available assessment data to maximize statistical power to detect any differences between treatments and to minimize the effect of missing outcome data. Difference in the rate of decline between minocycline (any dose) and placebo, and between patients allocated 400 mg and 200 mg of minocycline hydrochloride, was compared using a time-by-treatment interaction test, with time modeled as a continuous variable. Comparisons of time prescribed trial medication over the 24-month follow-up period split by treatment groups are displayed in Kaplan-Meier curves, with statistical significance determined by log-rank tests. Reasons for stopping trial medication and adverse events are tabulated by treatment group. We used SAS, version 9.3, software (SAS Institute) for statistical analyses. All *P* values were from 2-sided tests and results were deemed statistically significant at *P* < .05. The trial is registered with the International Standard Randomized Clinical Trials Number register (ISRCTN16105064) and the European Union Clinical Trials Register (EudraCT 2013-000397-30).

## Results

**Figure 1.**
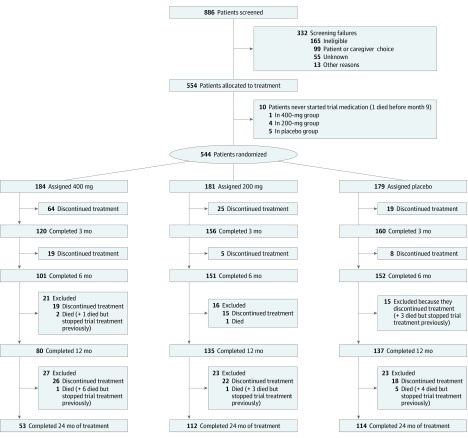
Flowchart of Study

Between May 23, 2014, and April 14, 2016, a total of 886 patients were screened for eligibility, from whom 554 participants entered the trial, from 32 National Health Service memory services in England and Scotland. The reasons for screening failures are given in eTable 2 in Supplement 2. Ten patients did not start trial medication and, as prespecified in the protocol, were excluded from all analyses (Figure 1). The baseline characteristics of the 544 eligible participants were well balanced across treatment groups (Table 1). We obtained sMMSE assessments for 542 of the 544 participants (99.6%) at baseline, 498 of 544 participants (91.5%) at 6 months, 453 of 537 participants (84.4%) at 12 months, 420 of 528 participants (79.5%) at 18 months, and 403 of 517 participants (77.9%) at 24 months (eTable 1 in Supplement 2).

**Table 1.** Baseline Characteristics by Treatment Allocation for the 544 Eligible Patients

Characteristic	Patients No. (%)		
	Minocycline Hydrochloride, 400 mg (n = 184)	Minocycline Hydrochloride, 200 mg (n = 181)	Placebo (n = 179)
Age, y			
<65	22 (12.0)	22 (12.2)	21 (11.7)
65-74	68 (37.0)	66 (36.5)	66 (36.9)
≥75	94 (51.1)	93 (51.4)	92 (51.4)
Age, mean (SD), y	74.3 (8.0)	74.1 (8.4)	74.6 (8.1)
Sex			
Male	104 (57)	100 (55.2)	99 (55.3)
Female	80 (43)	81 (44.8)	80 (44.7)
Race/ethnicity, No./total No. (%)			
White	173/183 (94.5)	169/176 (96.0)	171/176 (97.2)
Asian	5/183 (2.7)	1/176 (0.6)	3/176 (1.7)
Black	5/183 (2.7)	5/176 (2.8)	2/176 (1.1)
Other	0	1/176 (0.6)	0
Home circumstance			
Living with spouse, partner, or relative	153 (83.2)	153 (84.5)	149 (83.2)
Living alone	31 (16.8)	28 (15.5)	29 (16.2)
Duration of symptoms, mo			
<6	20 (10.9)	20 (11.0)	20 (11.2)
≥6	164 (89.1)	161 (89.0)	159 (88.8)
Duration of symptoms, mean (SD), mo	23.5 (18.3)	23.1 (17.8)	24.2 (18.0)
sMMSE score^a^			
24-26	100 (54.3)	97 (53.6)	96 (53.6)
27-30	84 (45.7)	84 (46.4)	83 (46.4)
sMMSE score, mean (SD)^a^	26.4 (1.9)	26.5 (1.9)	26.4 (1.8)
BADLS score^b^			
0-4	100/183 (54.6)	110 (60.8)	92/178 (51.7)
5-14	70/183 (38.3)	57 (31.5)	69/178 (38.8)
≥15	13/183 (7.1)	14 (7.7)	17/178 (9.6)
BADLS score, mean (SD)^b^	5.6 (6.3)	4.9 (5.4)	5.5 (5.5)

Abbreviations: BADLS, Bristol Activities of Daily Living Scale; sMMSE, Standardised Mini-Mental State Examination.

^a^ Scores range from 0 to 30, with higher scores indicating better cognitive function.

^b^ Scores range from 0 to 60, with higher scores indicating greater impairment.

**Figure 2.**
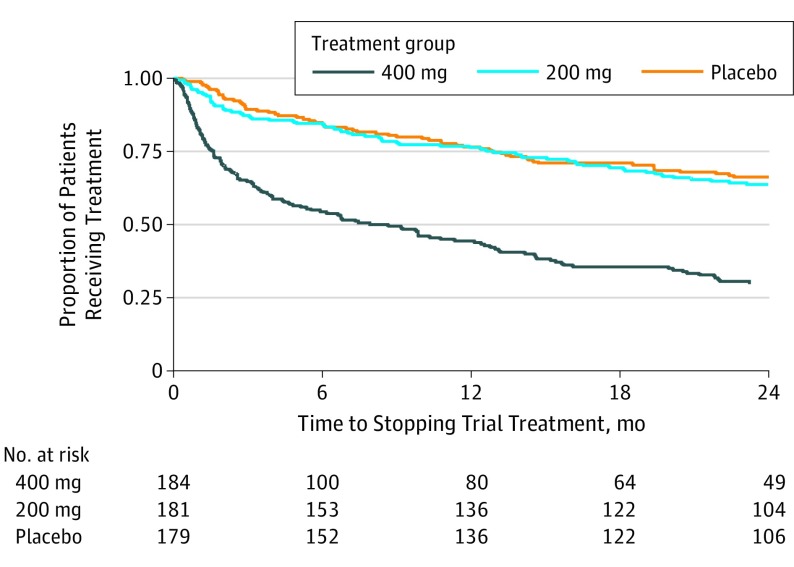
Proportion Taking Trial Treatment Over Time Minocycline hydrochloride, 400 mg, vs placebo: hazard ratio, 3.09; 95% CI, 2.27-4.20; *P* < .001; minocycline hydrochloride, 200 mg, vs placebo: hazard ratio, 1.11; 95% CI, 0.78-1.57; *P* = .56. Mean treatment duration for each group: minocycline hydrochloride, 11.4 months in the 400-mg group; minocycline hydrochloride, 18.6 months in the 200-mg group; and 18.9 months in the placebo group.

Minocycline hydrochloride, 400 mg, was poorly tolerated, with 28.8% of participants (53 of 184) completing 2 years of treatment, significantly fewer than in the 200-mg group (61.9% [112 of 181]) or the placebo group (63.7% [114 of 179]; *P* < .001) (Figure 1 and Figure 2). Minocycline hydrochloride, 200 mg, was well tolerated, with similar discontinuation rates with placebo. The mean duration of treatment was 11.4 months in the 400-mg group, 18.6 months in the 200-mg group, and 18.9 months in the placebo group. When reasons for stopping trial treatment were compared (Table 2), more participants allocated to minocycline than to placebo stopped because of gastrointestinal symptoms (42 in the 400-mg group, 15 in the 200-mg group, and 10 in the placebo group; *P* < .001), dermatologic adverse effects (10 in the 400-mg group, 5 in the 200-mg group, and 1 in the placebo group; *P* = .02), and dizziness (14 in the 400-mg group, 3 in the 200-mg group, and 1 in the placebo group; *P* = .01). Discontinuation rates did not differ by age, sex, or duration of symptoms (eTable 4 in Supplement 2).

**Table 2.** Incidence and Severity of Adverse Effects, Reasons for Stopping Treatment, and Serious Adverse Events by Treatment Allocation^a^

Characteristic	Minocycline Hydrochloride, 400 mg (n = 184)	Minocycline Hydrochloride, 200 mg (n = 181)	Placebo (n = 179)	Minocycline vs Placebo *P* Value
**Adverse Effects, No.**				
Dermatologic symptoms (hyperpigmentation, photosensitivity, rash)				
Mild	33	38	22	.04
Moderate	27	29	13	.008
Severe	1	2	3	.37
Gastrointestinal symptoms (diarrhea, nausea, sore mouth, vomiting)				
Mild	52	55	55	.74
Moderate	46	24	17	.004
Severe	6	1	4	.81
Neurologic symptoms (headache, visual or auditory disturbances, dizziness)				
Mild	53	57	51	.69
Moderate	27	16	16	.32
Severe	5	6	3	.36
Infections (oral or genital candidiasis, vaginitis, anal irritation, bacterial enteritis, staphylococcal, or *Clostridium difficile*)				
Mild	16	10	16	.46
Moderate	17	17	25	.10
Severe	4	4	7	.25
**Reasons for Stopping Trial Treatment, No.**				
Gastrointestinal symptoms (reflux, constipation, diarrhea, gastroenteritis)	42	15	10	<.001
Dizziness	14	3	1	.01
Dermatologic symptoms (rash, hyperpigmentation, photosensitivity)	10	5	1	.02
Hematologic symptoms	5	3	1	.16
Impaired renal function	2	5	4	.81
Infection	1	2	2	.74
Shortness of breath	6	0	0	.08
Worsening dementia	1	3	3	.57
Depression or anxiety	4	2	2	.63
Joint or muscle pain	2	0	2	.47
Concomitant disease or illness	9	6	7	.91
General deterioration in physical health	2	0	2	.47
Unknown	1	0	0	.48
Unspecified adverse effect	5	2	7	.17
Patient or carer choice	23	21	18	.49
Total	127	67	60	<.001
**Serious Adverse Events, No.**^b^				
Gastrointestinal	3	8	10	.14
Respiratory	8	8	10	.54
Falls and fractures	6	11	13	.21
Endocrine and metabolic	2	1	9	.002
Cancer	12	3	11	.30
Hematologic or thrombosis	3	1	2	.98
Dermatologic	0	1	0	.48
Stroke	4	5	12	.02
Psychiatric symptoms and seizures	6	8	4	.33
Cardiocirculatory	14	9	11	.94
Renal	3	2	2	.81
Infection	10	1	19	<.001
Other	7	11	2	.03
Total	78	69	105	<.001

^a^ Differences were compared by χ^2^ test with associated 2-sided *P* values.

^b^ Serious adverse events are adverse events that were fatal (10 in the 400-mg group, 6 in the 200-mg group, and 12 with placebo), lifethreatening, resulted in or prolonged hospital admission, or resulted in disability (further information in eTable 5A and eTable 5B in Supplement 2).

Because of the higher treatment withdrawal rate, fewer assessments were obtained for the 400-mg treatment group than for the 200-mg and placebo groups (eTable 1 in Supplement 2). At 24 months, 68.4% (119 received out of 174 expected) of patients in the 400-mg group, 81.8% (144 of 176) of patients in the 200-mg group, and 83.8% (140 of 167) of patients in the placebo group provided sMMSE assessments.

**Figure 3.**
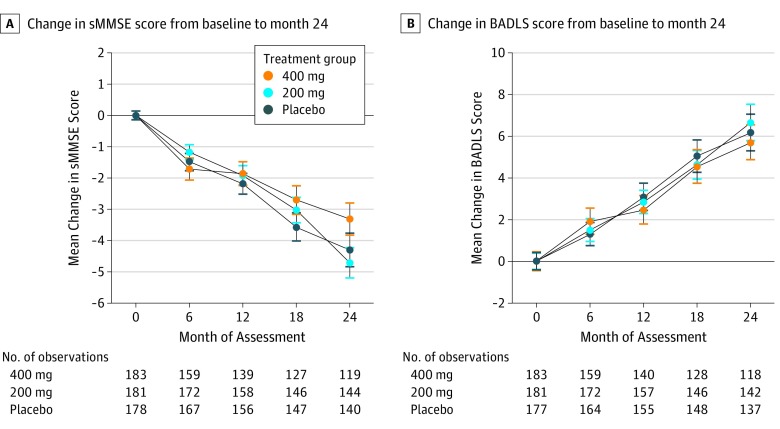
Change in Mean (SE) Standardised Mini-Mental State Examination (sMMSE) and Bristol Activities of Daily Living Scale (BADLS) Scores From Baseline to Month 24 A, Change in sMMSE score from baseline to 24 months. Any dose of minocycline vs placebo: treatment effect = 0.07; 95% CI, –1.1 to 1.2; *P* = .90; 400 mg vs 200 mg: treatment effect = 1.17; 95% CI, –0.1 to 2.5; *P* = .08. B, Change in BADLS score from baseline to 24 months. Any dose of minocycline vs placebo: treatment effect = –0.53; 95% CI, –2.4 to 1.3; *P* = .57; 400 mg vs 200 mg: treatment effect = –0.31; 95% CI, –0.2 to 1.8; *P* = .77. Baseline scores are set to zero (baseline sMMSE scores: 26.3 in the 400-mg group, 26.5 in the 200-mg group, and 26.4 in the placebo group; baseline BADLS scores: 5.6 in the 400-mg group, 4.9 in the 200-mg group, and 5.5 in the placebo group). Treatment effect is the estimated difference in 2-year decline from repeated measures analyses; *P* values are from tests comparing rate of decline between groups (time by treatment interaction) from repeated measures analyses. Results from intention-to-treat analysis of 554 patients.

Change from baseline in sMMSE over time is shown in Figure 3A. There was a mean 4.1-point reduction in the combined minocycline groups over 24 months compared with 4.3 points in the placebo group. The combined minocycline group had a mean sMMSE score 0.1 points higher than the placebo group (95% CI, −1.1 to 1.2; *P* = .90). The decrease in mean sMMSE scores over 24 months was less in the 400-mg group than in the 200-mg group (3.3 vs 4.7 points), but this difference was not significant (treatment effect = 1.2; 95% CI, −0.1 to 2.5; *P* = .08).

Worsening of BADLS scores over 24 months was similar in all groups: 5.7 in the 400-mg group, 6.6 in the 200-mg group, and 6.2 in the placebo group, with no significant differences between participants receiving minocycline compared with those in the placebo group (treatment effect = −0.53; 95% CI, −2.4 to 1.3; *P* = .57) or between those allocated 400 mg and those allocated 200 mg of minocycline (treatment effect = −0.31; 95% CI, −0.2 to 1.8; *P* = .77) (Figure 3B).

Participants in the 400-mg group who stopped treatment were similar to those in other groups, although they tended to be older (eTable 4 in Supplement 2). To assess how the higher number of missing outcome assessments in the 400-mg group than in the 200-mg or placebo groups (eTable 1 in Supplement 2) might have affected outcome comparisons, we performed sensitivity analyses to investigate potential bias from nonrandom withdrawal. There were 41 participants who had a baseline sMMSE assessment but no further assessments, so they did not contribute any information to the primary analysis (eFigure 1 in Supplement 2). Those who discontinue treatment in AD trials are often atypical, usually having worse cognitive and functional ability than those who continue treatment.^21^ This finding is evident from the scores of the 41 participants with a 6-month sMMSE assessment but no later assessments. The mean decrease in sMMSE score from baseline to 6 months in this subset was 3.9 points, a rate of decrease 3 times higher than the 1.3-point mean decrease among the 498 patients who had a 6-month sMMSE assessment and completed later assessments. It seems likely, therefore, that patients with no postbaseline assessments, who do not contribute to the estimate of the rate of decline, also had worse than average decline in cognitive and functional ability.

To estimate what effect the missing outcome data from the 41 participants with no postbaseline assessments might have had on the trial results, our sensitivity analyses made 2 different assumptions. In method 1, we assumed that, for the first 6 months, they declined at a rate of 3.9 points (as did those who had a 6-month sMMSE assessment but no further assessments) and then declined at the mean rate of 1.1 points every 6 months for the rest of the trial. Method 2 assumed that patients with no postbaseline assessments declined at the mean rate of those with assessments (ie, 1.3 sMMSE points for the first 6 months and 1.1 points every 6 months subsequently). The results from imputation method 1 and imputation method 2 are shown in eFigure 2 in Supplement 2. The results are not qualitatively different from those of the primary analyses. The only borderline significant (treatment effect = 1.2; 95% CI, 0.0-2.4; *P* = .05) differences seen in these sensitivity analyses were between the groups receiving 400 mg and 200 mg minocycline hydrochloride. However, because the 400-mg group had results a little better than the placebo group and the 200-mg group had results a little worse than the placebo group, and no difference between any dose of minocycline and placebo, this is likely a chance finding.

Because return rates for BADLs were also lower for the 400-mg group, we performed similar sensitivity analyses. There were 39 participants with no BADLS assessment after baseline who did not contribute to the primary analysis. Imputation method 1 assumed that their BADLS score worsened (ie, increased) by 3.7 points during the first 6 months and then by 1.9 points every 6 months for the rest of the trial. Method 2 assumed that their BADLS score worsened by 1.5 during the first 6 months and then by 1.9 points subsequently. Because BADLS is only valid for community-resident patients, scores for those receiving residential care were only imputed up until the last time point before moving into a care facility. The results for imputation methods 1 and 2 are shown in eFigure 3 in Supplement 2. Again, the results were not qualitatively different from those from the primary analyses of BADLS.

To investigate whether the efficacy of minocycline varied by baseline characteristics, we did subgroup analyses of change in sMMSE score over 24 months for minocycline (any dose) vs placebo by duration of symptoms, baseline sMMSE score, age, and sex (eFigure 4 in Supplement 2). There was no indication of any benefit from minocycline for those with shorter or longer duration of symptoms, lower or higher baseline sMMSE score, or for men vs women. There was a borderline significant trend toward greater efficacy in younger patients than in older patients, but this unanticipated finding could be a chance occurrence given the number of subgroup investigations.

In total, there were 252 reported serious adverse events, with the most common categories being neuropsychiatric and cardiocirculatory (Table 2). The number of serious adverse events was somewhat higher in the placebo group (n = 105) than the 400-mg group (n = 78) or 200-mg group (n = 69). Given that gastrointestinal symptoms were the main reason for stopping trial treatment, it is reassuring that the numbers of gastrointestinal serious adverse events in the minocycline groups were low and no higher than in the placebo group. Similarly, although more skin-related toxic effects, particularly pigmentation, were reported with minocycline than placebo (35.6% [130 of 365] vs 21.2% [38 of 179]; *P* < .001), few stopped trial treatment because of such toxic effects (Table 2), and only 6 skin toxic effects were considered severe (3 receiving any dose of minocycline and 3 receiving placebo). There were no differences in the numbers of patients stopping treatment because of impaired renal function, which had been a prior concern, nor in the numbers of renal serious adverse events. Twenty-eight patients died during the study: 10 who received 400 mg of minocycline hydrochloride, 6 who received 200 mg of minocycline hydrochloride, and 12 who received placebo (eTable 3 and eFigure 5A in Supplement 2). Fifteen of these 28 patients had stopped trial treatment prior to dying. One additional patient died without starting trial treatment. Rates of admission to residential care facilities were low in this population of patients with mild AD, with no difference in the numbers between trial groups (eFigures 5B and 5C in Supplement 2).

## Discussion

The MADE trial showed that, for patients with mild AD, 24 months of minocycline treatment at the doses tested does not delay the progress of cognitive or functional impairment, as measured by the widely used sMMSE and BADLS clinical rating scales. The trial also established that minocycline hydrochloride at a dose of 400 mg is poorly tolerated in this population, with fewer than one-third of participants completing 24 months of treatment. By contrast, 200 mg per day of minocycline hydrochloride was well tolerated.

The failure of minocycline treatment to slow the progression of cognitive and functional decline in patients with mild AD is disappointing given the evidence suggesting that neuroinflammation is instrumental in AD progression^7^ and given minocycline’s anti-inflammatory and neuroprotective effects, as well as the positive data from experimental AD models.^10,11,12,13,14,15,16^ Nonsteroidal anti-inflammatory drugs similarly failed to slow AD progression in clinical trials,^22^ despite long-term use being associated with a lower risk of developing AD in observational studies^23^ and promising data from transgenic models.^24^ Our findings parallel those of trials of minocycline in other neurodegenerative disorders in which, despite preclinical research suggesting neuroprotection, minocycline worsened outcomes in amyotrophic lateral sclerosis (with faster amyotrophic lateral sclerosis functional scale decline compared with placebo)^25^; had no effect in Huntington disease,^26^ multiple system atrophy,^27^ and negative symptoms of schizophrenia^28^; and only short-term benefits in multiple sclerosis.^29^

We consider that there could be 3 broad potential explanations for the negative results of our trial. First, although there is good evidence for neuroinflammation in AD,^7^ this may be a reaction to pathologic characteristics of the disease rather than an important factor in neurodegeneration, particularly in patients whose AD is mild. Second, even if neurodegeneration is accelerated by neuroinflammation, minocycline at the doses administered in the MADE trial may not have had sufficient activity to show efficacy. Animal studies, from which much of the evidence for minocycline as an anti-inflammatory and anti-AD agent come, generally used higher doses of minocycline (typically equivalent to 3-7 g per day in humans),^30^ and so it could be that trial participants were not exposed to a sufficiently high dose. We included the 400-mg group to investigate whether a higher dose enhanced efficacy. A study in amyotrophic lateral sclerosis^25^ that escalated doses from 200 to 400 mg reported that adverse events were unrelated to minocycline dose. The MADE trial established that treatment with 400 mg is poorly tolerated in patients with AD, with no apparent benefit from the higher dose, despite a mean treatment duration of about 1 year. Hence, efficacy of minocycline could not be enhanced by using higher doses.

Minocycline is potentially neuroprotective through anti-inflammation activity (suppression of microglial proliferation and activation, reduced IL-1β and IL-6 and tumor necrosis factor, decreased chemokine expression, and decreased metalloproteases), as well as antiapoptotic and antioxidant effects.^10,11,12,13,14,15,16^ A study in traumatic brain injury found reduced microglial activation, visualized with carbon 11–labeled PBR28 positron emission tomography,^31^ after 12 weeks of treatment with 200 mg of minocycline hydrochloride per day, indicating that the doses in the MADE trial can have a measurable effect on inflammation. The association between minocycline-sensitive microglial activation and neurodegeneration may, however, be complicated. Minocycline treatment in the traumatic brain injury study^31^ was also associated with increased plasma neurofilament light. The faster progression seen with minocycline in amyotrophic lateral sclerosis^25^ also suggests that some activated microglia might have a reparative function so that their inhibition could accelerate neurodegeneration. Our results do not suggest that minocycline worsens neurodegeneration in AD.

A third plausible explanation for the negative results of the MADE trial could be that minocycline did have some efficacy against AD, but treatment effects were too small to be detectable. It is difficult to discount this possibility. The MADE trial was, however, powered to detect minimal clinically important differences between minocycline and placebo, so smaller differences might not be considered of clinical relevance.

### Strengths and Limitations

Our pragmatic trial had a number of strengths. It was based within a broad network of academic and National Health Service memory services, and the wide eligibility criteria facilitated the recruitment of participants, many of whom had physical comorbidities and were representative of patients with mild AD. Outcome measures were limited in number, easy to administer reliably by trial staff, and chosen because any differences between minocycline and placebo would have unambiguous clinical relevance.

The potential limitations of the study include that biomarkers were not used to confirm AD diagnosis, because these and *APOE* genotyping are not routinely available within the National Health Service. Nonetheless, no diagnoses were revised by responsible clinical teams during the study, and the rates of decline were as expected in a population of individuals with mild AD and comparable between the most and least mildly impaired participants (eFigure 6 in Supplement 2). Adherence was also problematic, with few patients in the 400-mg group completing 2 years of treatment and only moderate adherence in the 200-mg and placebo groups.

Although the trial protocol specified that outcome assessments should be obtained irrespective of treatment compliance, that could not always be achieved despite the efforts of the trial team. Consequently, differential follow-up rates could have biased our results. However, despite the large number of treatment withdrawals in the 400-mg group and the consequent loss to follow-up of some participants, the results were essentially unchanged in sensitivity analyses investigating potential bias from missing data. These analyses indicate that bias from missing assessments would tend to underestimate decline, and more so in the 400-mg group than in the 200-mg and placebo groups, because more assessments were missing from the 400-mg group. We are consequently confident that we have not missed a benefit of 400 mg of minocycline because of missing data.

## Conclusions

Two years of minocycline treatment for patients with mild AD does not result in any clinically meaningful difference in the rate of decline of cognitive and functional ability. This finding is disappointing but robust.

## Notes

Supplement 1. **Trial Protocol**

Supplement 2. **eTable 1.** Follow-up Rates for sMMSE and BADLS by Treatment Allocation and Follow-up Period **eTable 2.** Reasons for Failed Screens Into MADE Trial **eTable 3.** Causes of Death **eTable 4.** Baseline Characteristics of Those Who Stopped MADE Treatment **eTable 5A.** Line by Line Listings of Serious Adverse Events (SAEs) Categorised **eTable 5B.** Serious Adverse Events Categorised by Treatment Allocation and Whether on Treatment (IMP) **eFigure 1.** Flow Chart: Follow-up Completeness Over Time **eFigure 2.** Change in sMMSE From Baseline to Month 24 (A) Using Imputation Method (1) and (B) Using Imputation Method (2) to Estimate Scores for Patients With No Follow-up Past Baseline **eFigure 3.** Change in BADLS From Baseline to Month 24 (A) Using Imputation Method (1) and (B) Using Imputation Method (2) to Estimate Scores for Patients With No Follow-up Past Baseline **eFigure 4.** Subgroup Analyses of Change in sMMSE Over 24 Months for Minocycline (Any Dose) Versus Placebo by Baseline Characteristics **eFigure 5.** Probability of (A) Survival, (B) Remaining Community Resident and (C) Being Alive and Community-Resident by Treatment Allocation: Kaplan-Meier Survival Plots **eFigure 6.** Average Decline of sMMSE Split by Baseline sMMSE Score of 24-26 or 27-30

Supplement 3. **Data Sharing Statement**
