# Supplementary material for: Minocycline at 2 Different Dosages vs Placebo for Patients With Mild Alzheimer Disease: A Randomized Clinical Trial
Source: JAMA Neurol. 2019 Nov 18;77(2):164–74. doi: 10.1001/jamaneurol.2019.3762 (PMC6865324; doi:10.1001/jamaneurol.2019.3762)
Supplement: Supplement 1. — Trial Protocol [file jamaneurol-77-164-s001.pdf]

CLINICAL STUDY PROTOCOL

Minocycline in Alzheimer's disease efficacy trial: the MADE Trial

Version 12: 22nd March 2018

Previous Versions: v1, 3rd February 2013; v2, 15th April 2013; v3, 22nd May 2013; v4: 24th July 2013; v5, 23 Aug 2013; v6, 02 October 2013; v7, 08 January 2014, v8, 23 January 2014, V9, 09 June 2014; V10: 23rd October, 2015, V11:30th June 2016..

IRAS Project ID: 117466
EudraCT number: 2013-000397-30

MREC number: 13/EE/0063

ISRCTN number: TBC

Funding: EME Grant EME 11/47/01

Trial Co-Sponsors
King's College London, London and South London and the Maudsley NHS Foundation Trust
Sponsor Contact: Helen Critchley, King's Health Partners Clinical Trials Office, 16th Floor, Guy's Tower, London SE1 9RT
Tel: 020 7188 5732
Fax: 0207188 8330
Email: helen.critchley@kcl.ac.uk

SIGNATURES

Chief Investigator
Name: Professor Robert Howard (Professor of Old Age Psychiatry and Psychopathology)
Address: Department of Old Age Psychiatry
Institute of Psychiatry, PO Box 070, De Crespigny Park, London SE5 8AF
Telephone: + 44 (0)20 7848 0545
Fax: +44 (0)20 7848 0632
Email:robert.j.howard@kcl.ac.uk

Chief Investigator's signature **Date**
Print name:

Principal Investigator
Name:
Address:

Telephone:
Fax:
E-mail:

Principal Investigator's signature **Date**
Print name:

SUMMARY

Minocycline in Alzheimer's disease efficacy trial: The MADE Trial

Alzheimer's disease (AD) is a major public health issue with approximately 700,000 people in the UK suffering from dementia of whom some 400,000 have Alzheimer's disease. With the population aging, the incidence of Alzheimer's disease is projected to increase with an estimated one million affected by 2020, at a cost of £20 billion a year. The imperative to discover and develop treatments that can stop or at least delay disease progression is clear. None of the drug treatments licensed for AD have been shown to affect progression of the illness and, despite a better understanding of the pathogenesis of AD, clinical trials of potentially disease modifying treatments so far undertaken have had disappointing results.

There is a substantial body of evidence to indicate that minocycline may be neuroprotective in neurodegenerative diseases such as AD. Although the primary neuroprotective target of minocycline in the central nervous system is not known, the principal effects of minocycline include: inhibition of microglial activation, attenuation of apoptosis and suppression of the production of reactive oxygen species. Minocycline is arguably the most promising off-patent candidate for AD modification that is not currently in trials and it is cheap and well tolerated.

MADE is a multi-centre, randomised, controlled trial in very mild AD, which primarily aims to determine whether minocycline is superior to placebo in affecting the disease course, over a 2-year period, as measured by reduced rate of decline in cognition (Standardised Mini-Mental State Examination (sMMSE)) and function (Bristol Activities of Daily Living Scale (BADLS). MADE will also compare the safety and tolerability of minocycline at doses of 200mg and 400mg per day. MADE aims to randomise 560 participants in a semi-factorial (2x1) design between minocycline (400mg), minocycline (200mg) or placebo minocycline.

To make widespread participation feasible, MADE trial procedures and documentation are kept to a minimum. Patients may enter the study on stable anticholinesterase or memantine treatment and may commence or discontinue such treatment during the course of the study. The MADE outcome measures (sMMSE and BADLS) are clinically relevant, widely used in routine clinical practice, have good psychometric properties and have been shown to be sensitive to change in previous AD clinical trials.

RESPONSIBLE PERSONNEL

Trial Management Group (TMG)

**Psychiatry**	
**South London and Maudsley *NHS* Foundation *Trust***	
**Robert Howard (Chief Investigator)**<br>Professor of Old Age Psychiatry and Psychopathology,<br>Department of Old Age Psychiatry,<br>Institute of Psychiatry,<br>Box 070, De Crespigny Park,<br>London SE5 8AF.<br>Tel: 020 7848 0545<br>Email: robert.j.howard@kcl.ac.uk	**Simon Lovestone (Co-Investigator)**<br>Professor of Old Age Psychiatry<br>Director of Research,<br>King's Health Partners<br>NIHR Biomedical Research Centre for<br>Mental Health, King's College London<br>Institute of Psychiatry<br>De Crespigny Park, London SE5 8AF<br>Tel: 020 7848 0239<br>Email: simon.lovestone@kcl.ac.uk
**Clive Ballard (Co-Investigator)**<br>Professor of Age Related Diseases,<br>King's College London,<br>Wolfson Centre for Age Related Diseases,<br>Guy's Campus,<br>King's College London, SE1 1UL<br>Tel: 02078488054<br>Email: clive.ballard@kcl.ac.uk	**Suzanne Reeves (Co-Investigator)**<br>Clinical Senior Lecturer<br>Department of Old Age Psychiatry,<br>Institute of Psychiatry, Box 070,<br>De Crespigny Park,<br>London SE5 8AF.<br>Tel: 020 7848 0548<br>Email: suzanne.j.reeves@kcl.ac.uk
**Manchester Mental Health and Social Care Trust**	
**Iracema Leroi (Principal Investigator)**<br>Consultant in Old Age Psychiatry<br>Clinical Senior Lecturer in Psychiatry<br>Institute for Brain, Behaviour and Mental Health<br>University of Manchester<br>Jean McFarlane Building, 3rd floor<br>Oxford Rd, Manchester M13 9PL<br>Tel: 01613 067942<br>Iracema.Leroi@manchester.ac.uk	**Alistair Burns (Co-Investigator)**<br>Professor of Old Age Psychiatry, Hon.<br>Consultant Psychiatrist,<br>The University of Manchester,<br>Institute for Brain, Behaviour and Mental<br>Health<br>Jean McFarlane Building, 3rd floor<br>Oxford Rd, Manchester M13 9PL<br>Tel: 0161 306 7942<br>Email: alistair.burns@manchester.ac.uk
**Oxford Health NHS Foundation Trust**	
**Rohan Van Der Putt (Principal Investigator)**<br>Consultant in Old Age Psychiatry<br>Thames Valley DeNDRoN,<br>Room 4401D, Level 4<br>John Radcliffe Hospital<br>Headington, Oxford OX3 9DU<br>Tel: 01865 231556<br>Email: rohan.vanderputt@oxfordhealth.nhs.uk	**Gordon Wilcock (Co-Investigator)**<br>Emeritus Professor of Geratology<br>University of Oxford<br>Level 4, John Radcliffe Hospital<br>Headington, Oxford<br>UK OX3 9DU<br>Tel: 07734 297334<br>Email: gordon.wilcock@ndcn.ox.ac.uk
**Rupert McShane (Co-Investigator)**<br>Consultant Old Age Psychiatrist,<br>Oxford Health NHS Foundation Trust,<br>Warneford Hospital,<br>Oxford, OX3 7JX<br>Tel:01865 741717<br>Rupert.McShane@oxfordhealth.nhs.uk	

Kent and Medway NHS and Social Care Partnership Trust	
**Richard Brown (Principal Investigator)**<br>Kent and Medway NHS and Social Care Partnership Trust,<br>Sittingbourne Memorial Hospital<br>Bell Rd, Sittingbourne,<br>Kent ME10 4DT<br>Tel: 01795 438446 / 01795 418359<br>Email: richard.brown@kmpt.nhs.uk	**Ananth Puranik (Co-Investigator)**<br>Clinical Director<br>Kent and Medway NHS and Social Care Partnership Trust,<br>35 Kings Hill Avenue,<br>Kings Hill, West Malling Kent, ME19 4AX<br>Tel: 01622726899<br>Email: ananth.puranik@kmpt.nhs.uk
**Kompancariel Kuruvilla (Co-Investigator)**<br>Kent and Medway NHS and Social Care Partnership Trust<br>Elmstone Unit<br>Thanet Mental Health Unit<br>Margate<br>CT9 4 BF<br>Tel: 01843 234584<br>Email: kompancariel.kuruvilla@kmpt.nhs.uk	
**Norfolk and Suffolk NHS Foundation Trust**	
**Chris Fox (Co- Investigator)**<br>Clinical Senior Lecturer in Psychiatry,<br>Norwich Medical School,<br>University of East Anglia,<br>Norwich Research Park,<br>Norwich, NR4 7TJ<br>Tel: 01603 593583<br>Email: chris.fox@uea.ac.uk	**Judy Rubinsztein (Principal Investigator)**<br>Consultant Psychiatrist<br>Wedgwood House, OPMHS,<br>West Suffolk Hospital<br>Hardwick Lane<br>Bury St Edmunds, IP33 2QZ<br>Tel: 01284 71955/ 07539240283<br>Email: Judy.Rubinsztein@nsft.nhs.uk
**Birmingham and Solihull Mental Health NHS Foundation Trust**	**Camden and Islington NHS Foundation Trust**
**Peter Bentham (Principal Investigator)**<br>Consultant in Working Age Dementia, Birmingham and Solihull Mental Health NHS Foundation Trust, The Barberry, 25 Vincent Drive Birmingham B15 2FG<br>Tel: 0121 301 2070Email:<br>peter.bentham@bsmhft.nhs.uk	**Gill Livingston (Principal Investigator)**<br>Professor of Psychiatry of Older people, University College London<br>Hon Consultant psychiatrist, Camden and Islington NHS Foundation Trust.<br>Tel: 020 7561 4218<br>Email: g.livingston@ucl.ac.uk
**Cambridgeshire and Peterborough NHS Foundation Trust**	**West London Mental Health NHS Trust**
**John O'Brien (Principal Investigator)**<br>Foundation Professor of Old Age Psychiatry<br>Department of Psychiatry<br>University of Cambridge<br>Level E4, Box 189<br>Addenbrooke's Hospital<br>Hills Road<br>Cambridge CB2 0QQ<br>Tel: +44 (0)1223 760682<br>Email: john.obrien@medschl.cam.ac.uk	**Dr Bryan Corridan (Principal Investigator)**<br>West London Cognitive Disorders Treatment & Research Unit, Lakeside Mental Health Unit,<br>West Middlesex University Hospital Site,<br>Twickenham Road,<br>Isleworth, TW7 6AF<br>Tel: 0208 483 1827<br>Email: Bryan.Corridan@wlmht.nhs.uk

**Vanya Johnson (Co-Investigator)**<br>Consultant Psychiatrist<br>Older Peoples Mental Health<br>Newtown Centre,<br>Nursery Road,<br>Huntingdon, PE29 3RJ<br>Tel: (Mob) 0773 263 5517<br>Email: vanya.johnson@cpft.nhs.uk	
**Sussex Partnership NHS Foundation Trust**	**Berkshire Healthcare NHS Foundation Trust**
**Naji Tabet (Principal Investigator)**<br>Senior Lecturer in Old Age Psychiatry, Postgraduate Medicine, Brighton and Sussex Medical School, Mayfield House, University of Brighton, Brighton BN1 9PH.<br>Tel: 01892 603107.<br>Email: n.t.tabet@brighton.ac.uk	**Dr Nicholas Woodthorpe (PI)**<br>Consultant Psychiatrist<br>Wokingham Hospital<br>41 Barkham Road<br>Wokingham<br>Berkshire RG41 2RE<br>Tel: 01189495101<br>Email:Nick.woodthorpe@berkshire.nhs.uk
**Northamptonshire Healthcare NHS Foundation Trust**	**Leicestershire Partnership NHS Trust**
**Paul Koranteng (Principal Investigator)**<br>Consultant Psychiatrist<br>Northamptonshire Healthcare NHS Foundation Trust<br>Berrywood Hospital<br>Berrywood Drive<br>Duston<br>Northampton, NN5 6UD<br>Tel: 01604 682636<br>Email: paul.koranteng@nhft.nhs.uk	**Dr Santanu Chakrabarti (PI)**<br>Consultant Psychiatrist<br>The Evington Centre, Leicester General Hospital,<br>Gwendolen Rd<br>Leicester LE5 4QG<br>Tel: 01162255964<br>Email: santanu.chakrabarti@leicspart.nhs.uk
**Northumberland, Tyne & Wear NHS Foundation Trust**	**Surrey and Borders Partnership NHS Foundation Trust**
**Robert Barber (Principal Investigator)**<br>Consultant Old Age Psychiatrist / Hon Clinical Senior Lecturer<br>Northumberland, Tyne & Wear NHS Foundation Trust<br>Older Peoples' Mental Health Services<br>Centre for the Health of the Elderly<br>Campus for Ageing and Vitality<br>(Formerly Newcastle General Hospital)<br>Newcastle upon Tyne , NE4 6BE<br>Tel: **0191 246 8776**<br>Email: robert.barber@ntw.nhs.uk	**Ramin Nilforooshan (Principal Investigator)**<br>Consultant Psychiatrist<br>Medical Lead for R&D<br>Brain Science research unit<br>ACU, Holloway Hill, Lyne, Chertsey, Surrey KT16 0AE<br>Tel: 01932 722 444<br>Email: Ramin.nilforooshan@sabp.nhs.uk
**Coventry & Warwickshire Partnership NHS Trust**	**Bradford District Care Trust**
**Dr Demi Onalaja (Principal Investigator)**<br>Consultant in Old Age Psychiatry<br>The Caludon centre,<br>Clifford Bridge Road, Walsgrave<br>Coventry, CV2 2TE<br>Tel:- 024 7696 8153<br>Fax:- 024 7696 7975<br>Email:Oluwademilade.Onalaja@covwarkpt.nhs.uk	**Dr.Anilkumar Pillai (PI)**<br>Consultant psychiatrist,<br>Older people's Mental Health Unit,<br>Daisy Hill House, Lynfield Mount Hospital,<br>Heights lane,<br>Bradford BD9 6DP,<br>Tel:01274 228549<br>Email: Anilkumar.Pillai@bdct.nhs.uk,

Ayrshire and Arran NHS	St George's Healthcare NHS
**Dr Ajay Macharouthu (Principal Investigator)**<br>Consultant in Liaison Psychiatry for elderly<br>20 Lister Street<br>Crosshouse Hospital<br>Kilmarnock<br>KA2 0BE<br>Tel 07745287906<br>Email: ajayverma.macharouthu@nhs.net	**Dr Jeremy Darryl Isaacs (PI)**<br>Consultant Neurologist<br>St George's Healthcare NHS Trustnorth<br>Department of Neurology<br>Blackshaw Road<br>London SW17 0QT<br>Tel: 0208 725 4630 | 07867 785211<br>Fax: 0208 725 4700<br>Email: jeremy.isaacs@nhs.net
**South Staffordshire and Shropshire Healthcare NHS Foundation Trust**	**Leeds and York Partnership NHS Foundation Trust**
**Dr Ejaz Nazir (Principal Investigator)**<br>Consultant Old Age Psychiatry<br>Department of Old Age Psychiatry,<br>The Redwoods Centre,<br>Shrewsbury<br>SY3 8DS<br>Tel: 01743 210000<br>Email: ejaz.nazir@sssft.nhs.uk	**Dr Wendy Neil (Principal Investigator)**<br>Consultant Psychiatrist<br>Leeds and York Partnership Foundation<br>Linden House<br>St Mary's Hospital<br>Greenhill Rd<br>Leeds<br>LS12 3QE<br>Tel: 01138556217<br>Email: wendyneil@nhs.net
**Lincolnshire Partnership NHS Foundation Trust**	**The Royal Bournemouth and Christchurch Hospitals NHS Foundation Trust**
**Dr Ban Alkaissy (Principal Investigator)**<br>Manthorpe Centre<br>Grantham Hospital<br>101 Manthorpe Road<br>Grantham<br>NG31 8DG<br>Tel: 01476 578901<br>Email: ban.alkaissy@sssft.nhs.uk	**Dr Divya Tiwari (PI)**<br>Acute Stroke Unit<br>Castle Lane East<br>BH7 7DW<br>Bournemouth<br>Tel: +4401202 705387<br>Email: Divya.Tiwari@rbch.nhs.uk
**Solent NHS Trust**	**Cornwall partnership Foundation trust (CFT)**
**Dr Kayode Osanaiye (PI)**<br>Consultant Psychiatrist in Old Age<br>Older Persons Mental Health, St James Hospital,<br>Langstone Centre, Locksway Road, Portsmouth.<br>Hampshire. PO4 8LD.<br>Tel: 02392684683.<br>E-mail: kayode.osanaiye@solent.nhs.uk	**Dr Vandana Mate**<br>Consultant old age Psychiatrist DR<br>Banham House,<br>Bodmin Hospital site,<br>Boundary Road,<br>Bodmin,<br>Cornwall PL31 2QT<br>Phone number: 01208 834340<br>vandana.mate@cft.cornwall.nhs.uk
**2gether NHS Foundation Trust**	**Cumbria Partnership NHS Foundation Trust**
**Dr Tarun Kuruvilla (PI)**<br>Consultant in Old Age Psychiatry<br>Hon Sen Clinical Lecturer - University of Bristol;<br>Dementia Research Champion – Glos R&D Consortium;<br>Charlton Lane Centre, Cheltenham, Glos. GL53 9DZ;	**Dr Marisa Wray**<br>Consultant in old age psychiatry<br>Garburn House,<br>Westmorland General Hospital,<br>Kendal<br>LA9 7RG

Tel: 01242 634462<br>Email: Tarun.Kuruvilla@glos.nhs.uk	Tel: 01539 715009<br>marisa.wray@cumbria.nhs.uk
**Derbyshire Healthcare NHS Foundation Trust**	**Nottinghamshire Healthcare NHS Trust**
**Dr Simon Thacker (PI)**<br>Consultant Psychiatrist,<br>Clinical Director<br>Centre for Dementia<br>Ashbourne Centre<br>Kingsway, Derby<br>De22 3LZ<br>Tel: 01332623700 ext 33683<br>simon.thacker@derbyshcft.nhs.uk	**Dr Sujata Das (PI)**<br>Consultant Psychiatrist,<br>Nottinghamshire Healthcare NHS Trust<br>Duncan Macmillan House<br>Porchester Rd<br>Nottingham,<br>NG3 6AA<br>Tel: 01623622515 ext 3387<br>Email: sujata.das@nottshc.nhs.uk
**Avon and Wiltshire Mental Health**	**South West London and St George's Mental Health NHS Trust**
**Dr Rosalind Ward**<br>Consultant Psychiatrist and Medical Lead for Later Life, North Somerset<br>Windmill House,<br>Windmill Road, Kenn,<br>Clevedon, BS21 6UJ<br>Tel: 01275 335300 M: 07776304197<br>rosalind.ward@nhs.net	**Dr Robert Lawrence**<br>CMHT & Clinical Research Unit (CRU/POAN)<br>Barnes Hospital<br>South Worple Way<br>London SW14 8SU<br>Tel: 020 3523 3680<br>Email: robert.lawrence@swlstg-tr.nhs.uk
**Black Country Partnership NHS Foundation Trust**	**Southend University Hospital NHS Foundation Trust**
Dr **Tarik Qassem**<br>Consultant in Old Age Psychiatry<br>Black Country Partnership NHS Foundation Trust<br>Email: T.Qassem@warwick.ac.uk	Dr Farhad Huwez<br>Consultant Physician and Geriatrician<br>Department of Medicine for Elderly<br>Southend University Hospital NHS Trust<br>Prittlewell Chase/Westcliff-On-sea<br>SS0 0RY<br>Tel: 01702435555 ext 5052<br>Email: Farhad.Huwez@southend.nhs.uk
**Statisticians**	**Study Pharmacist**
**Rosie Bradley, Richard Gray**<br>Clinical Trial Service Unit (CTSU),<br>Richard Doll Building,<br>Old Road Campus,<br>Roosevelt Drive, Oxford OX3 7TF.<br>Tel: 01865 743537<br>Email:richard.gray@ctsu.ox.ac.uk	**Nigel Barnes**<br>Director of Pharmacy & Medicines Management<br>Birmingham & Solihull Mental Health Foundation Trust Central Pharmacy<br>Venture House, 355 Slade Road<br>Erdington, Birmingham B23 7JA<br>Tel: 0121 301 5179<br>E mail: nigel.barnes@bsmhft.nhs.uk
Service User Representatives	
TBC via Alzheimer's Society	TBC via Alzheimer's Society

Data Monitoring Committee and Ethics Committee (DMEC)

**Professor Peter Crome (Chairman)**<br>Department of Primary care and Public Health, University College London<br>E mail: p.crome@ucl.ac.uk	**Dr Jeremy Brown (Independent Physician)**<br>Consultant Neurologist, Addenbrooke's Hospital Memory Clinic, Cambridge<br>E mail: jmb75@medschl.cam.ac.uk
**Professor Sarah Walker (Independent Statistician)**<br>MRC Clinical Trials Unit, 125 Kingsway, London, WC2B 6NH, UK<br>**Email:** sarah.walker@ctu.mrc.ac.uk	

Trial Steering Committee (TSC)

**Professor Gordon Wilcock (Chairman)**<br>Professor of Geratology, University of Oxford<br>E mail: Gordon.wilcock@ndm.ox.ac.uk	**Professor Declan McLoughlin (Independent Physician)**<br>St Patrick's Hospital, Dublin<br>E mail: d.mccloughlin@tcd
**Professor Robert Howard (Chief Investigator)**<br>(see previous)	**Professor Richard Gray (Trial Statistician)**<br>(see previous)
**Consumer Representative** -TBC	

MADE Study Office and Randomisation

**MADE Study Office**<br>Oxford Clinical Trial Service Unit (CTSU), Richard Doll Building, Old Road Campus, Roosevelt Drive, Oxford, OX3 7LF<br>**Randomisation**<br>Telephone: 0800 585323 (toll free in UK)<br>Tel: 01865 765615 (answering machine outside office hours);<br>Fax: 01865 743986;	
**Trial Manager (Oxford)**<br>Linda Kelly<br>Tel: 01865 743507<br>E-Mail: linda.kelly@ctsu.ox.ac.uk	**Project Manager (Kings)**<br>Olga Zubko<br>Tel: 020 7848 5923<br>E-Mail: olga.zubko@kcl.ac.uk
**Trial Administrator (Oxford)**<br>Emma Harper<br>Tel: 01865 765615<br>E-Mail: emma.harper@ctsu.ox.ac.uk	**Data Manager (Oxford)**<br>Lynn Pank<br>Tel: 01865 743507<br>E-Mail: lynn.pank@ctsu.ox.ac.uk

TABLE OF CONTENTS

Signatures..P2
Summary…P3
Responsible Personnel…P4
Abbreviations..P10

1) Background and Rationale..P12

2) Trial Objectives and Design...P13

2.1. Trial Objectives...P13
2.2 Trial Design...P13
2.3 Ethical Considerations...P14

3) Outcome Measures...P15

3.1. Primary Efficacy Parameters...P15
3.2 Safety...P16

4) Patient Entry...P16

4.1 Recruitment and Screening for eligibility.. P16
4.2 Randomisation..P17

5) Treatment and Follow-up Procedures...P17

5.1 Trial Treatment..P17
5.2 Details of Investigational Medicinal Product (IMP)
5.3 Unblinding..P19
5.4 Other Treatments (Concomitant Medication)..P19
5.5 Treatment Compliance..P20
5.6 Drug Accountability..P20
5.7 Continued Treatment..P20
5.8 Assessments..P20
5.9 Minimising Loss to Follow-up..P21
5.10 Expected Duration of Study..P22

6) Safety Monitoring Procedures...P23

6.1 Specification, Timing and Recording of Safety Parameters..P23
6.2 Procedures for Recording and Reporting Adverse Events..P23
6.3 Reporting Responsibilities..P24
6.4 Adverse Events of Special Interest..P25

7) Sample Size, Statistics and Data Management...P25

7.1 Sample Size..P25
7.2 Statistical Analysis..P2
7.3 Data Management..P26

8) Organisation...P27

8.1 Local Principal Investigator..P27
8.2 Local Study Coordinator..P27
8.3 Trial Steering Committee..P28
8.4 Data Monitoring and Ethics Committee..P28
8.5 Ethics & Regulatory Approvals..P28
8.6 Quality Assurance..P29
8.7 Publication Policy ..P29
8.8 Financial Aspects..P30

9) References...P31

10) Appendices (separate document)

Appendix 1: Standardised Mini-Mental State Examination (sMMSE)
Appendix 2: Bristol Activities of Daily Living Scale (BADLS)

Appendix 3: NIA/AA Criteria for Probable and Possible Alzheimer's Disease
Appendix 4: MADE Patient Information Sheet
Appendix 5: MADE Caregiver Information Sheet
Appendix 6: MADE Patient Consent Form
Appendix 7: MADE Caregiver Consent Form
Appendix 8: MADE Personal Legal Representative Consent Form
Appendix 9: MADE GP Letter
Appendix 10: MADE Follow-up form
Appendix 11: MADE SAE form

Abbreviations

AD – Alzheimer's disease.
AE – Adverse Event.
ALS – Amyotrophic Lateral Sclerosis
AR – Adverse Reaction.
BADLS – Bristol Activities of Daily Living Scale.
CI – Chief Investigator.
CRA - Clinical Research Associate.
CRF – Case Report Form.
CTSU - Clinical Trial Service Unit
DeNDRoN – Dementia and Neurodegenerative Diseases Research Network.
DSUR - Development Safety Update Report.
DMEC – Data Monitoring and Ethics Committee.
EME - Efficacy and Mechanism Evaluation Programme .
GCP – Good Clinical Practice.
HTA - Health Technology Assessment .
IMP - Investigational Medicinal Product .
KCL – Kings College London.
KHP-CTO – Kings Health Partners Clinical Trials Office.
LSC – Local Study Coordinator.
MADE - Minocycline in Alzheimer's Disease Efficacy trial.
MHRA – Medicines and Healthcare Products Regulatory Agency.
MHRN – Mental Health Research Network.
MMSE – Mini-Mental State Examination.
MR – Modified Release.
NIA/AA – National Institute on Aging / Alzheimer's Association.
NRES – National Research Ethics Service.
PI- Principal Investigator.
R & D – Research and Development.
REC - Research Ethics Committee.
RW – Research Worker.
SAE – Serious Adverse Event.
SAR – Serious Adverse Reaction.
SUSAR – Suspected Unexpected Serious Adverse Reaction.
SLE – Systemic Lupus Erythematosus.
sMMSE – Standardized Mini-Mental State Examination.
SOP – Standard Operating Procedures.
SSA – Site Specific Assessment.
TM – Trial Manager.
TMG – Trial Management Group.
TSC – Trial Steering Committee.
UAR – Unexpected Adverse Reaction.

1) BACKGROUND AND RATIONALE

There is a substantial body of evidence to indicate that minocycline may be neuroprotective in neurodegenerative diseases. Although the primary neuroprotective target of minocycline in the central nervous system is not known, the principal effects of minocycline include inhibition of microglial activation, attenuation of apoptosis and suppression of the production of reactive oxygen species (Plane et al 2010). In animal models of brain ischaemia, minocycline has demonstrated protection (Yrjanheikki et al 1999; Carty et al 2008; Lechpammer et al 2008) and worsening (Tsuji et al 2004) of hypoxic-ischaemic injury and reduced ischaemia-induced behavioural deficit (Liu et al 2007). In stroke patients, open-label treatment with 200mg/day of minocycline for 5 days after infarct has been reported to improve functional outcome (Lampl et al 2007). In animal models of Parkinson's disease, studies have reported both reduced microglial activation and neuronal death (Du et al 2001; Radad et al 2010) and reduced microglial activation and worsened neuronal death (Diquet et al 2004; Yang et al 2003). Pilot clinical trials in Parkinson's disease at a dose of 200mg/day over 18 months have shown no effect on symptoms and no significant increase in adverse events (NINDS NET-PD Investigators 2008).

Minocycline treatment in the superoxide dismutase1 transgenic mouse model for amyotrophic lateral sclerosis (ALS) delayed the onset of neurodegenerationand muscle strength decline (Zhu et al 2002). A completed phase III trial in ALS, however, reported worse outcomes with minocycline in terms of faster decline in forced vital capacity and manual muscle strength (Gordon et al 2007). Suggested explanations for this are that the dose of up to 400mg/day used may have contributed to fatigue in a highly susceptible population, and that increased glutamate receptor 1 phosphorylation may have promoted glutamate toxicity to motor neurons (Huntington Study Group 2004). In AD, both in vitro and in vivo studies have reported reduced microglial activation, attenuated neuronal death, astrogliosis and improved behavioural performance (Hunter et al 2004; Familian et al 2006; Familian et al 2007; Seabrook et al 2006; Cuello et al 2010; Ryu et al 2006; Choi et al 2007; Parachikova et al 2010; Noble et al 2009). There have, however, been no published clinical trials to date in AD patients and none are currently registered as recruiting.

The minimum daily dose of minocycline that offers neuroprotection in the human has not been established. A dose of 200mg/day is generally very well tolerated in the long term treatment of acne (Goulden 1996) and has been shown to be neuroprotective in acute stroke (Lampl et al 2007), spinal cord injury (Casha et al 2009) and multiple sclerosis (Metz et al 2009). However, 200mg/day of minocycline, although well tolerated, did not improve outcomes in trials in Parkinson's disease (NINDS NET-PD Investigators 2008) or Huntington's disease (Bonelli et al 2004). Some authors have argued that one reason for the failure of some trials may be that such doses are too low to be neuroprotective, pointing out that the typical effective dose in animal studies would be equivalent to 3 to 7g/day in humans (Plane et al 2010). It would not be feasible or ethical to subject AD participants to such very high doses of minocycline, but MADE includes a comparison of 400mg/day vs 200mg/day to investigate the tolerability of 400mg/day and whether the higher dose confers increased efficacy.

Alzheimer's disease is a major public health issue and the imperative to discover and develop treatments that can stop or at least delay disease progression is clear. Symptomatic AD treatments in the form of cholinesterase inhibitors and memantine have been the mainstay of current treatment for more than 10 years, but do not slow progression of the disease. With a more detailed understanding of the basic biology of the AD process, a wide

range of cellular and animal model systems have been developed within which several candidate disease-modifying treatments appear promising, though no such agent has performed successfully in phase III trials (for notable recent examples see Green et al 2009; Aisen et al 2011). Unfortunately, the development of treatments for AD is a complex and difficult process. Slowness of the neurodegenerative process and the substantial difficulties involved in demonstrating that this has been changed by treatment are major contributors to this problem. Minocycline is arguably the most promising off-patent candidate for AD modification that is not currently in trials and is cheap and well tolerated. The time is now absolutely right for an adequately powered clinical trial, conducted for a sufficiently long period to demonstrate efficacy on simple cognitive and functional outcomes. The results, even if clearly negative, will move the field on to a significant degree. If minocycline treatment does not influence cognitive and functional change over 2 years, this will end serious interest in the drug as an AD treatment. But, if differences greater than those considered to represent minimum clinically important differences for AD therapies are seen, this will rapidly pave the way for Phase III effectiveness trials supported by the Health Technology Assessment (HTA) programme.

2) TRIAL OBJECTIVES AND DESIGN

2.1. Trial Objectives

MADE is a multi-centre randomised controlled trial with the following objectives:

The primary objective is to determine whether minocycline is superior to placebo in slowing the disease course of early AD, over a 2-year period, measured by reduced rate of decline in:

- **(i)** Cognition (sMMSE)
- **(ii)** Function (BADLS)

The secondary objectives of **MADE** are:

- **(i)** To compare the safety and tolerability of minocycline at doses of 400mg/day and 200mg/day
- **(ii)** To determine whether 400mg/day offer superior neuroprotection to 200mg/day.
- **(iii)** To investigate associated risks of side-effects and serious adverse events.
- **(iv)** To estimate the magnitude of any statistically significant positive treatment effects on cognitive and functional decline and thereby inform the design and powering of a future phase III trial of definitive clinical effectiveness within the NHS.

2.2 Trial Design

MADE is a pragmatic, Phase II, 3-arm randomised, double-blind, multicentre study, with a semi-factorial design.

Participants will be allocated to one of three arms:

Arm 1- Minocycline 400mg/day

Arm 2- Minocycline 200mg/day

Arm 3- Placebo.

Trial treatment will continue for a two year period.

Randomisation will be carried out centrally by the Oxford Clinical Trial Service Unit (CTSU) by telephone (0800 585 323, toll free in UK), email (randomisation@ctsu.ox.ac.uk) or fax (01865 743986). A minimised randomisation algorithm will be used to balance allocations across four factors: centre, duration of symptoms prior to randomisation (<6 months, ≥6 months), baseline sMMSE score (24-26, 27-30), and age (<65, 65-74, ≥75 years). This will reduce the risk of chance imbalances between arms with respect to known prognostic factors. Patients, caregivers, clinicians, outcome assessors and investigators will be blinded to treatment allocation.

2.3 Ethical Considerations

Any neuroprotective benefit from minocycline is likely to outweigh the risks. Minocycline is routinely used at doses of 200mg per day in the long-term treatment of acne and is considered safe in this indication (Goulden 1996). *Rare* side effects include acute renal failure, irreversible skin pigmentation, and *very rarely* systemic lupus erythematosus (SLE).

We will apply for multi-centre research ethics approval and local research governance approval for the studies. The study personnel, the co-investigators and collaborators as the management group and independent Trial Steering Committee (TSC) will ensure that the study is conducted within appropriate NHS and professional ethical guidelines. Information will be kept strictly confidential and held in accordance with the Data Protection Act (1998). Data will be held on a secure database on a password-protected university computer. Access to data will be restricted to the research team.

Participation in **MADE** carries only a 1 in 3 risk of randomisation to placebo and patients will not have to forgo treatment with a cholinesterase inhibitor or memantine if their responsible clinician considers that they would benefit from such treatment.

Potentially eligible patients will be approached by a clinician, who knows them, and given the opportunity to hear more about research activities. The study may also be advertised directly to patients via posters, leaflets etc. Those individuals who are interested in learning more about research will be referred to a member of the research team who will provide an information sheet with full study details including possible benefits and risks. They will be offered the opportunity to ask questions and discuss any queries with the carer/relative/doctor and make a date for the eligibility interview. Interested patients will be sent the information sheet with the invite to screening and written informed consent obtained prior to commencing the screening assessment.

All researchers will be trained in gaining consent and in informed consent through the Dementia and Neurodegenerative Diseases Research Network (DeNDRoN) or Mental Health Research Network (MHRN) training course. National Research Ethics Service (NRES) Guidance on the content and format of patient information and consent documentation will be followed. Consumer representatives will be involved to ensure that these documents and all other written trial materials are fit for purpose. Information about the study will be mailed to potential participants and their caregivers.

The main potential ethical issue in dementia trials is that the disease may interfere with an individual's ability to give informed consent. Because we are studying people with mild dementia, most of the participants will have capacity to give informed consent for their involvement. Consequently, fully informed, written, consent will be obtained from patients entering the MADE study. However as patients will remain in the study for up to two years it is likely that some may lose capacity over this period. In light of this, patients will be also asked what they would want to happen in the event of them losing capacity during the

course of the study. They will be given the option of either withdrawing at this point or a decision being made on their behalf by their Personal Legal Representative in line with The Medicines for Human Use (Clinical Trials) Regulations 2004. This person would most likely be the patient's main carer, who would have best knowledge of the individual's attitudes and stated preference to research and consequently best placed to judge whether they would have wished to continue if they had capacity. In this situation the patient's agreement to participate will still be obtained to their best level of understanding and they will not remain in the study if they refuse or show significant distress.

3) OUTCOME MEASURES

3.1. Primary Efficacy Parameters

Short, simple outcome measures that are already widely used in routine clinical practice will be used to measure changes in the two core defining features of dementia due to AD, cognition and functional ability.

(i) The first primary outcome measure is the standardised Mini-Mental State Examination (sMMSE) (Molloy et al 1991; Molloy et al 1997), a widely used clinician-rated instrument for assessing cognition. Scores range from 0 to 30 with higher scores indicating better cognitive function.

The original Mini-Mental State (MMSE) was designed as a brief test to detect organic brain disease and quantify the degree of cognitive impairment. It is still probably the most widely used cognitive test in the world (Folstein et al, 1975) and has good psychometric properties (Tombaugh and McIntyre (1992). The sMMSE was developed to provide raters with explicit guidelines for administration and scoring with the aim of improving reliability of the instrument. The sMMSE differs from the MMSE in four main areas: serial sevens are omitted, the order of the time orientation questions is changed, for all questions a response time limit imposed and for each item unambiguous scoring rules are given. The sMMSE score is considered to be of clinical relevance with the minimum clinically important difference estimated to be 1.4 points (Howard et al 2010). The sMMSE has been shown to be sensitive to the effects of anti-dementia drug treatment in previous AD clinical trials (AD2000 Collaborative Group 2004; Feldman et al 2001; Howard et al 2007).

(ii) The second outcome measure is the Bristol Activities of Daily Living Scale (BADLS; Bucks et al 1996) used to assess **Functional ability (activities of daily living)** Scores range from 0 to 60 with higher scores indicating greater impairment.

The BADLS was specifically designed for use with dementia patients living in the community and participating in clinical trials. The BADLS is sensitive to change, correlates well with economic outcomes and despite being a carer rated instrument appears to have good test-retest reliability. The levels of disability between which the scale aims to discriminate were also carer generated giving some perspective on the value of change with the minimum clinically important difference estimated to be 3.5 points (Howard et al 2010). The BADLS has also been shown to be sensitive to change across a wide range of functional disability in previous AD clinical trials (AD2000 Collaborative Group 2004; Howard et al 2012).

Data will be collected on the sMMSE at Screening, 6, 12, 18 and 24 months, and for the BADLS at Baseline, 6, 12, 18 and 24 months.

3.2 Safety
Safety parameters including: blood monitoring of haematological, renal and hepatic function as well as documentation of skin reactions, gastrointestinal and neurological symptoms and concurrent infections (bacterial enteritis, clostridium difficile and orogenital candidiasis) will be assessed and recorded every 3 months.

Renal function monitoring:
MDRD formula will be used to calculate eGFR at baseline and changes in creatinine will be used to monitor renal function post baseline:

(i) Any patient with a follow up creatinine of ≥25% and <50% higher than their baseline value can remain on treatment but will have a repeat blood sample in 2-3 weeks. If creatinine remains the same or higher then a further check will be required. Any patient with a follow-up creatinine of ≥50% higher than baseline can remain on treatment but will have a repeat blood sample within 10 days. If creatinine remains the same or higher then study treatment will be stopped (unless an obvious alternative cause is identified eg, NSAID use, other illness)

4) PATIENT ENTRY

4.1 Recruitment and Screening for Eligibility
Patients with very mild (defined as sMMSE > 23 points) AD (by NIA/AA criteria, McKhann et al 2011) will be identified from memory services, both within the participating NHS Trusts, where the Principal Investigators practise and within the network of memory services supported by DeNDRoN. Potentially eligible patients will be approached by a clinician who knows them and provided with an opportunity to hear more about research. The JDR (Join Dementia Research) recruitment tool will also be used. Those interested will meet with a member of the research team who will provide further information about the study. Potentially interested individuals will be given an opportunity to review the information and will be offered an opportunity to ask questions over the telephone, and make a date for the interview. Written informed consent will be obtained prior to commencing the screening assessment for the trial and after they have received the information sheet.

Screening: The diagnosis and provisional eligibility for the study will first be confirmed (see inclusion/exclusion criteria below). The sMMSE will be performed and this score will also be used as the baseline value. Blood will be taken for analysis of full blood count and biochemical profile. The screening blood results and concomitant medicines must be reviewed and recorded to confirm eligibility before randomisation.

Inclusion criteria will be:

- Diagnosis of possible or probable AD by NIA/AA criteria (McKhann et al 2011).
- sMMSE score >23 with no upper limit.
- Giving informed consent to participate
- Aged 50+
- Participants must have a potential informant who will assist in the administration of the BADLS

Exclusion criteria will be:

- Known allergy to tetracycline antibiotics
- Female of childbearing potential. Patients must be surgically sterile (hysterectomy, bilateral salpingectomy / oophorectomy) for at least 6 months minimum or have undergone bilateral tubal occlusion / ligation at least 6 months prior or have been post-menopausal for at least 1 year
- Uncontrolled serious concomitant illness
- Known chronic kidney disease stages 3b-5
- Moderate liver disease (see Child-Pugh for Classification of Severity of Liver Disease)
- Abnormal serum chemistry laboratory value at Screening deemed to be clinically relevant by the investigator.
- Withholds consent for the study team to inform his/her GP
- Systemic Lupus Erythematosus (SLE).
- Participation in another Clinical Trial of an Investigational Medicinal Product (IMP) in the previous 28 days.

Contraindications, warnings and special precautions to minocycline use are not described further in the protocol and the investigator should refer to the Summary of Product Characteristics http://emc.medicines.org.uk/.

4.2 Randomisation

After informed consent to being randomised has been obtained, and baseline assessments completed, randomisation will be carried out centrally by the **Oxford Clinical Trial Service Unit (CTSU)** randomisation service (tel 0800 585323). The person randomising will need to answer **all** of the telephone questions and should complete the **MADE** randomisation form (Appendix C) before calling to help in preparing for them. Alternatively, randomisation forms may be faxed - or scanned and e-mailed - to the **MADE** randomisation service (fax 01865 743986, e-mail: randomisation@ctsu.ox.ac.uk) who will call back with a treatment allocation. After **all** the necessary details have been provided, two treatment pack numbers will be allocated and a MADE patient trial number allocated. The recruiting PI (or other medically qualified doctor with a substantive or honorary contract with the recruiting NHS Trust and who has signed the 'Recruiting Investigator site delegation of authority form') should complete a **MADE** prescription form (provided in the study folder). The trial prescription will assign the patient the two allocated numbered treatment packs, which make up a 13-week treatment of minocycline 400mg/day, 200mg/day or matching placebo. The bulk of the medicine will be stored at Polar Speed with batches of the IMP (quantity depending on pharmacy storage capacity) dispatched to the trial pharmacies. The MADE trial office will monitor stock levels and further batches of treatment packs will be sent automatically once stocks become low. Instruction leaflets for the trial treatments are provided, which should be given to the patient with a copy in the patient's clinical notes. The baseline assessments should be labelled with the patient's **MADE** trial number and posted or faxed to the **MADE** Study Office in Oxford. The patient's GP should be notified that they are in **MADE** and a specimen "Letter to GP" is provided for this purpose (Appendix I).

5. TREATMENT AND FOLLOW-UP PROCEDURES

5.1 Trial Treatment

Trial treatment will be oral minocycline modified release capsules or identically appearing placebo packed into treatment cartons sufficient for 13 weeks' treatment (with a small overage).

The dosing regimens for the 3 treatment arms are:
Arm 1 - Minocycline (400mg) - two 100mg MR capsules of minocycline in the morning and two 100mg MR capsules in the evening.
Arm 2 - Minocycline (200mg) – one 100mg MR capsule of minocycline plus one minocycline placebo capsule in a morning and one 100mg MR capsule of minocycline plus one minocycline placebo capsule in an evening.
Arm 3 - Placebo minocycline – two minocycline placebo capsules in a morning and two placebo minocycline capsules in the evening.
Treatment packs will be supplied on a 3-monthly basis for a total treatment duration of 24 months.

5.2 Details of Investigational Medicinal Product

Manufacture

MODEPHARMA is responsible for arranging the IMPs' manufacture as well as project management and assistance relating to the IMP for the trial including preparation of the IMPD. The actual manufacturing of placebo, all IMP packaging and labelling, and final QP release of the IMPs will be undertaken by Piramal Healthcare UK Ltd (Licence Number 29595).
Acnamino MR 100mg capsules will be used as the active treatment. These will be procured and supplied to Piramal Healthcare for IMP packaging by MODEPHARMA or a NHS pharmacy with a wholesale licence. Acnamino MR 100mg capsules are hard gelatin capsules each containing one pink film-coated tablet and one peach enteric-coated tablet. Each capsule contains 100mg of the active substance minocycline as minocycline hydrochloride. Placebo tablet intermediates will be made using similar tablet tooling and film coating colour to the tablets in the active Acnamino™ MR 100mg capsule. The blinding of the placebo product is achieved by using the same capsule size and similar gelatin capsule body and cap colours as the Acnamino™ MR 100mg capsule. Placebo blister strips and patient treatment packs will match those of the active substance, and will be labelled in the same way.

Packaging and Labelling

Both active and placebo IMP for the trial will be packaged under QP control by Piramal Healthcare UK Ltd. Capsules will be packed in blister strips of 27 capsules with a colour-coded Annex 13 compliant label. 7 blister strips (189 capsules) will be placed in a carton, each with its own individual randomisation number (or treatment pack number) and colour-coded Annex 13 compliant label. Patients will be given 2 cartons, each containing 7 blister strips, at randomisation and every 3 months subsequently. This will make up a 3-month (13 weeks) supply. Patients will be directed to take 1 capsule from one carton and 1 capsule from the other carton every morning and every evening. As the capsules and blisters will look the same, for patients' ease, the labels used on the first and second cartons (and blisters) will have two different colours.

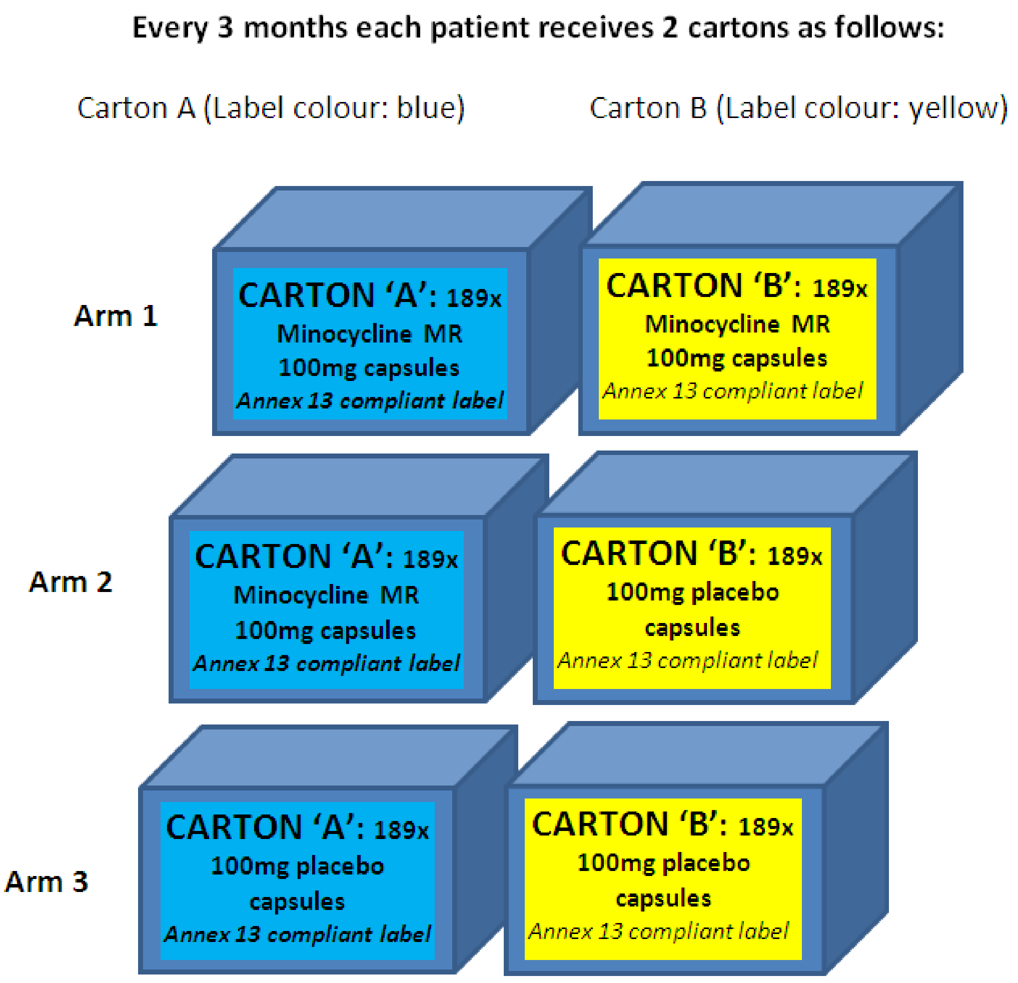

3-month supply of IMP

Storage and dispensing
Batches of treatment packs will be distributed to participating trial pharmacies by Polarspeed. Drug supplies must be kept in a secure, limited access storage area, in their original packaging and under the authorised storage conditions for the Acnamino 100mg MR capsules which are specified as "Store in the original package". Trial participants will be advised to store medication at ambient temperature, and out of the reach of children. All unused medication should be destroyed by the site pharmacy. Receipt, usage and destruction must be documented on the respective forms. Account must be given for discrepancies.

5.3 Unblinding

Investigators and patients will remain blinded to the treatment allocation throughout the trial. Unblinding should not normally be necessary as serious side-effects should be dealt with on the assumption that the patient is on active minocycline treatment. Study medication should be omitted rather than unblinded. Request for unblinding should be directed to the MADE Study Office (0800 585323) during office hours. If considered urgently necessary for patient management, the randomisation service can be telephoned to unblind trial treatments (0800 585323).

5.4 Other Treatments (Concomitant Medication)

Patients may be randomised into the MADE study whilst taking a cholinesterase inhibitor or memantine. Cholinesterase inhibitors and memantine may also be commenced or discontinued during the course of the study at the discretion of the responsible consultant.
Other concomitant medications which may interact with minocycline (listed in the Summary of the Product Characteristics (SmPc) for minocycline) will be recorded at each visit and the prescriber informed:

- ACE Inhibitors - absorption of minocycline decreased by quinapril tablets
- Antacids, adsorbants and vitamin/mineral supplements - absorption of minocycline is impaired by the concomitant administration of antacids, iron, calcium, aluminium, magnesium and zinc salts (interactions with specified salts, antacids and kaolin) unless taken 3 hours apart. Dosages should be maximally separated
- Antibacterials - minocycline should not be used with penicillins as minocycline can decrease its effectiveness.
- Anticoagulants - tetracyclines depress plasma prothrombin activity and reduced dosages of concomitant anticoagulants may be necessary
- Diuretics – may aggravate nephrotoxicity by volume depletion.
- Ergotamine and ergometrine – increased risk of ergotism.
- Retinoids - Administration of isotretinoin should be avoided shortly before, during and shortly after minocycline therapy as the combined administration of the two drugs increases the risk of benign intracranial hypertension.
- Ulcer healing drugs – absorption of minocycline is decreased by sucralfate and bismuth salts.

If side effects are reported their significance will be discussed with the study doctor. Depending upon severity, participant will be asked to continue with the study drug if possible and a review by the study doctor will be arranged in 2 weeks. If at the time of the review the side effects are ***severe enough to warrant withdrawal*** from the study, participants should be advised to omit the morning dose and a further review arranged in 2 weeks. If side effects persist, participants should be advised to take a temporary (eg 2-week) break from IMP treatment and will be able to re-start once the symptoms resolve. If side-effects persist, participants should be advised to stop taking the study drug. A MADE change of patient status form should be completed to document the reasons for stopping treatment. Participants will be able to re-start IMP at any point after the break should they wish to and if this decision is approved by the study clinician (PI). Participants should seek to re-start at half dose and after a couple of weeks try moving back up to full dose if possible but can remain on half dose for the duration of the trial if full dose is not well tolerated. Please note that patients who are on half dose will be dispensed IMP less frequently than those on full dose.

5.5 Treatment Compliance

Treatment compliance will be monitored by capsule count at the 6, 12, 18 and 24 month visits and monitored over the phone at week 2, and then at months 3, 9, 15 and 21. Participants should be asked to bring any unused study medication at each follow-up visit and at the end of the trial. Unused study medication will be obtained from the carer at these assessments. The local Principal Investigator (PI) or research worker will keep a log of

study medication returns, return date and amount of study medication returned and enter the information on the Case Report Form (CRF). Once returned medication has been logged, it should be destroyed by the local pharmacy. Carers will also be questioned regarding study drug compliance at all interim assessments. The study specific prescriptions will be maintained in the pharmacy file for audit purposes.

5.6 Drug accountability

The study drug will be dispensed by the local site pharmacy and full accountability of dispensing by pharmacy and of returns (at each face to face visit) will be undertaken by the PI or one of the research team. Pharmacy departments at each site will maintain a study medication dispensing log, including date dispensed, batch number, expiry date, and number of capsules dispensed. In addition the unique code numbers assigned to the treatment pack and trial patients will be recorded. The study specific prescriptions will be maintained in the pharmacy file for audit purposes. The investigator should verify that all unused or partially used drug supplies have been returned by the patients. Once unused medication has been logged, it can be destroyed by the local pharmacy.

5.7 Continued Treatment

Arrangements for continued treatment at the end of the trial will be made on an individual patient basis by the PI or other clinicians responsible for the patient's care at this point. Responsible clinicians will be asked to record on the last patient follow-up form what treatment plan is in place for the individual patient. On present evidence, no recommendation can be made about treatment beyond 2 years but the Data Monitoring and Ethics Committee (DMEC) will scrutinise the accumulating data from MADE and, if clear evidence for or against minocycline treatment emerges, will notify the TSC who will then make appropriate recommendations (see sections 8.3 and 8.4).

5.8 Assessments

Screening and Randomisation will be performed prior to the assessments described below.

Baseline: the baseline assessment must take place within 28 days of screening The BADLS will then be administered to complete the primary outcome assessments. Three months study drug will be supplied via the trial pharmacy.

2-week assessment: a telephone assessment of safety, tolerability, compliance and concomitant medicines will be made.

3, 9, 15 and 21 month assessments: an assessment of safety, tolerability, compliance and concomitant medicines will be made. Blood will be taken for analysis at the clinic or patient's home. Three months study drug will be supplied via the trial pharmacy.

6, 12 and 18 month assessments: compliance and safety will be assessed and concomitant medicines recorded. Primary outcomes will be administered and blood will be taken for analysis at the clinic or patient's home. Three months' study drug will be supplied via the trial pharmacy.

24 month assessment: primary outcomes will be administered. Compliance and safety will be assessed and concomitant medicines recorded.

Note that blood testing is not required for patients who are no longer taking MADE treatment but please continue to administer the other assessments including con-meds, SMMSE, BADLS, safety

For patients who have stopped the IMP AND follow-up but have not withdrawn their consent from the study, every attempt should be made to complete the SMMSE and BADLs at the 24 month visit. Where contact with the patient is not possible, an attempt to post the BADLS questionnaires and administer by telephone should be made.

Study Time- Event Chart

	Screen		Base	2/52	3/12	6/12	9/12	12/12	15/12	18/12	21/12	24/12
Diagnosis	*											
Inclusion	*											
Exclusion	*											
Con-meds	*			*	*	*	*	*	*	*	*	*
Consent	*											
Random		*										
SMMSE	*					*		*		*		*
BADLS			*			*		*		*		*
Dispense			*		*	*	*	*	*	*	*	
Compliance				*	*	*	*	*	*	*	*	*
Safety γ				*	*	*	*	*	*	*	*	*
FBC/Bio	*				*	*	*	*	*	*	*	

Assessments marked with large **bold stars** represent visits, those marked with small stars can be conducted over the telephone.

All visits have a permissible window of +- 7 days (telephone calls) and +- 14 days for face to face visits

Final, 24 months visits should be carried out +- 30 days within due date wherever possible but, if this is not possible, can be completed later to maximise completion rates for final assessments.'

γ Safety checks will include the documentation of skin reactions, gastrointestinal and neurological symptoms

Assessments during months 3, 9, 15 and 21 do not need to be performed for any patients that have stopped taking the IMP.

5.9 Minimising Loss to Follow-up

The trial aims to minimise the number of patients who discontinue treatment and, especially, the numbers with missing follow-up assessments. However, in some circumstances discontinuation may occur and can be initiated by the patient, their carers, investigators or other responsible physicians.

(i) Discontinuation from treatment only

Patients or their doctors commonly choose to discontinue clinical trial medication, e.g. because:

- The patient or carer withdraws consent to further treatment.
- Intercurrent illness or side-effects prevent further treatment.
- Change (or lack of change) in the patient's condition justify discontinuation of treatment in the clinician's opinion.

Most patients who discontinue treatment are happy to continue to be followed up. In this case, MADE outcome data should be collected in accordance with the protocol. The reason for stopping MADE treatment (e.g. side-effects, lack of perceived effectiveness, patient choice or other) and the use of other treatments (if any) should be recorded on the patient change of status form.

(ii) Discontinuation from treatment and follow-up assessments

Patients may choose to discontinue both treatment and study assessments. In this case, the local PI or research worker should attempt to ascertain the reason for a patient's discontinuation of follow-up assessments, without compromising their right to withdraw at any time without giving a reason, and record this on the patient change of status form. Note that, unless the patient specifically revokes their earlier consent for information about their progress to be sent to the MADE Study office, clinical information will continue to be collected and patient information will be retained in the trial database and used for intention-to-treat analyses of study outcome.

(iii) Loss to follow-up

A patient will be considered lost to follow-up if the investigator is not able to contact them despite multiple attempts. Every effort must be made; at least 2 telephone contacts plus 1 mailing should be documented. Loss to follow-up will be minimised by all available means, including use of centrally held NHS records, and will be monitored both locally and centrally. The site must discuss any loss to follow up case with the recruiting PI and the trial manager. Any patient who discontinues early from the study at any time must have their appropriate visit CRF pages completed. A patient will only be regarded as lost to follow-up with the agreement of the recruiting PI and the trial manager.

(iv) Patient transfers

For patients moving from the area, or to another doctor or hospital, every effort should be made for the patient to be followed up and for the other centre to take over responsibility for the patient assessments. A copy of the patient's study documentation will need to be sent to the new site, the patient will have to sign a new consent form and, until this occurs, the patient remains the responsibility of the original centre. The MADE Study office can help facilitate this process.

5.10 Expected Duration of Study

From the regulatory perspective, the end of the trial is defined as the end of the interventional phase, 24 months after the final patient is randomised. Completion for an individual patient is defined as completion of 24 months on the trial medication or discontinuation of follow-up for any reason. The trial may, however, be stopped earlier by the TSC if the Independent DMEC, in accordance with their charter, recommend to the TSC that the trial be stopped. The criteria for stopping the trial will be established as part of the standard operating procedures (SOP) of the DMEC (see section 8.4) at their first meeting.

6) SAFETY MONITORING PROCEDURES

6.1 Specification, Timing and Recording of Safety Parameters

Safety assessments will be made at the 6, 12, 18, and 24 month visits via a face to face interview. Blood will be taken for haematological and biochemical analysis including renal and hepatic function every 3 months for patients still taking MADE trial medication. The

researcher will also systematically enquire about changes in the patient's health state between assessments via the telephone interviews at 2 weeks, and either in person or via the telephone at 3, 9, 15, and 21 months.

6.2 Procedures for Recording and Reporting Adverse Events

The Medicines for Human Use (Clinical Trials) Regulations 2004 and Amended Regulations 2006 give the following definitions:

Adverse Event (AE): Any untoward medical occurrence in a subject to whom a medicinal product has been administered including occurrences which are not necessarily caused by or related to that product.

Adverse Reaction (AR): Any untoward and unintended response in a subject to an investigational medicinal product which is related to any dose administered to that subject.

Unexpected Adverse Reaction (UAR): An adverse reaction the nature and severity of which is not consistent with the information about the medicinal product in question set out in the summary of product characteristics (SPC) for that product (for products with a marketing authorisation) – available at http://emc.medicines.org.uk/ **Serious Adverse Event (SAE)**, **Serious Adverse Reaction (SAR)** or **Unexpected Serious Adverse Reaction**: Any adverse event, adverse reaction or unexpected adverse reaction, respectively, that:

- Results in death;
- Is life-threatening;
- Requires hospitalisation or prolongation of existing hospitalisation;
- Results in persistent or significant disability or incapacity;
- Consists of a congenital anomaly or birth defect.

A **Suspected Unexpected Serious Adverse Reaction** is usually referred to as a **SUSAR** and requires expedited reporting (see below).Note the term "severe" is often used to describe the intensity (severity) of a specific event. This is not the same as "serious," which is based on patient/event outcome or action criteria.

Assessment of Causality

The relationship between study drug and the SAE will be assessed by the local PI and categorised using clinical judgement into one of the following five categories:

Not related – temporal relationship not reasonable or event explained in isolation by another cause

Unlikely related – temporal relationship unlikely or event likely to be explained by another cause

Possibly related – temporal relationship is reasonable but event could be due to another equally likely cause

Likely related – temporal association is reasonable and event is more likely to be due to study drug than other cause

Definitely related – temporal relationship is reasonable and there is no other cause to explain event, or re-challenge is positive

For classification of causality ***possibly, likely*** *and* ***definitely related*** categories should be considered as reactions in the MADE trial.

The Investigator must report all SAEs, regardless of the causal relationship with the investigational medicinal products, to the Sponsor within 24 hours of awareness.

6.3 Reporting Responsibilities

King's College London (KCL) and South London and Maudsley NHS Foundation Trust (SLaM) as co-sponsors, have delegated the delivery of the Sponsor's responsibility for pharmacovigilance (as defined in Regulation 5 of the Medicines for Human Use (Clinical Trial Regulations 2004) to the King's Health Partners Clinical Trials Office (KHP-CTO). The list of expected adverse reactions contained in section 4.8 'Undesirable Effects' of the SmPC document will serve as the reference safety information, which will be used for the purposes of determining expectedness and thus SAE/SUSAR reporting.

The PI or other member of the research team should complete a SAE form (appendix 10.11) for all:

- SAEs,
- SARs and
- SUSARs

All SAEs, SARs and SUSARs (excepting those specified in this protocol as not requiring reporting) will be reported immediately (and certainly no later than 24hrs) by the Investigator to the KHP-CTO (fax: 020 7188 8330 or scan and e-mail to: jcto.pharmacovigilance@kcl.ac.uk) and CI (fax: 02078480632 or scan and e-mail to: robert.j.howard@kcl.ac.uk) for review in accordance with the current Pharmacovigilance Policy. The CI or delegate will then fax, or scan and e-mail, the form to the MADE Study Office. The MADE CI, or delegate, will review these events to determine whether they are SUSARS needing expedited reporting. In addition, AEs will be recorded and reviewed by MADE's independent DMEC at regular intervals. The KHP-CTO will report SUSARs to the Medicines and Healthcare Products Regulatory Agency (MHRA). The CI will delegate responsibility to the MADE Study Office at Oxford CTSU for reporting SUSARs and other SARs to the relevant ethics committees, PIs and R&D departments.

Reporting timelines:
SUSARs that are fatal or life-threatening must be reported to the MHRA and REC not later than 7 days after the Sponsor is first aware of the reaction. Any additional relevant information must be reported within a further 8 days.
SUSARs that are not fatal or life-threatening must be reported within 15 days of the Sponsor first becoming aware of the reaction.
The CI and KHP-CTO (on behalf of the co-sponsors), will submit a Development Safety Update Report (DSUR) relating to this trial IMP, to the MHRA and REC annually.

6.4 Adverse Events

Reporting Flowchart for SAEs, SARs and SUSARs

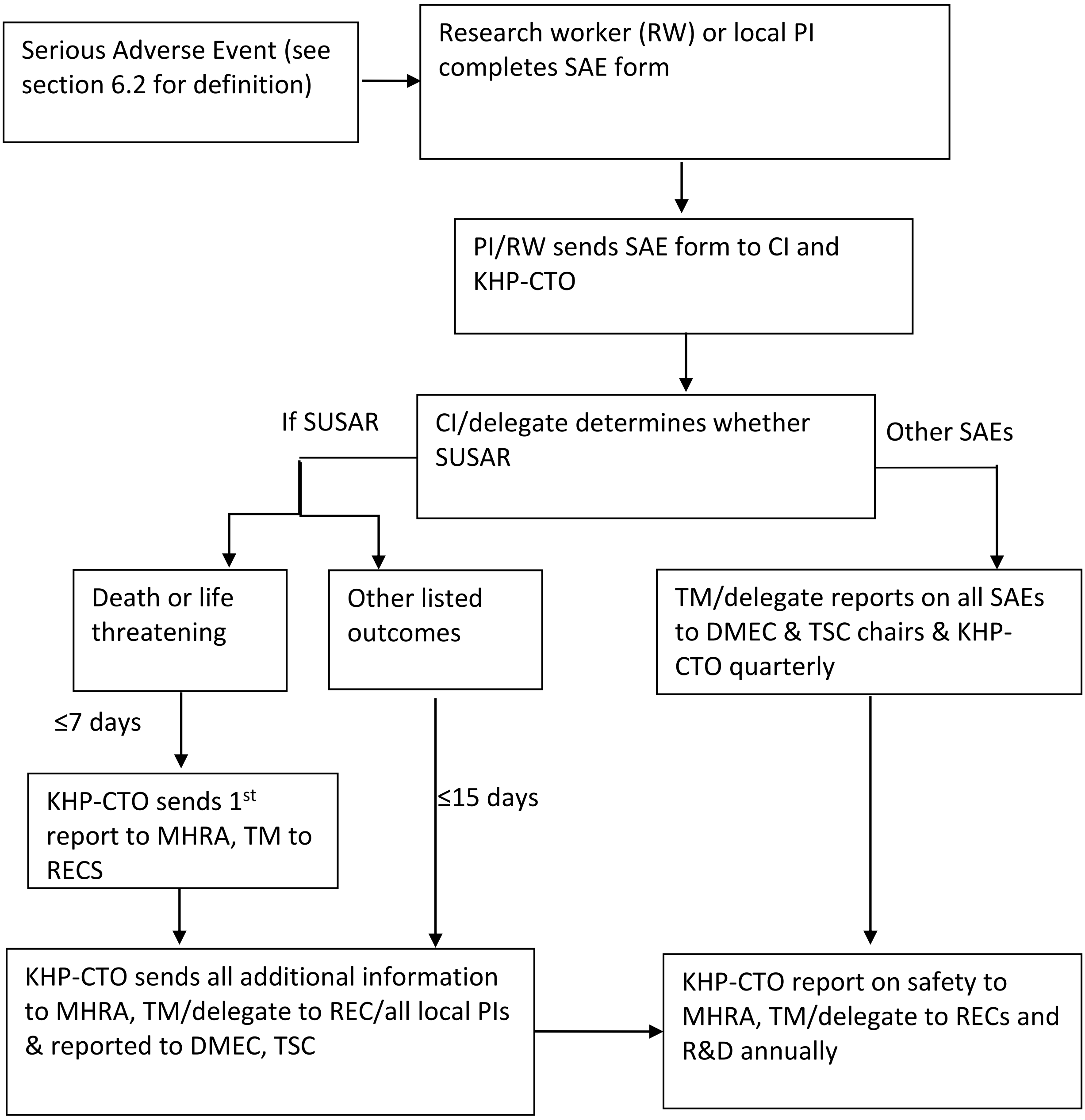

7) SAMPLE SIZE, STATISTICS, AND DATA MANAGEMENT

7.1 Sample Size

MADE aims to randomise at least 560 participants in a semi-factorial (2x1) design between minocycline (400mg), minocycline (200mg) or placebo minocycline. Even in mild AD, patient attrition is inevitable in a long term treatment trial and we estimate that by 2 years, about 15% of patients will have died, a further 5% will be in institutional care and 40% will have been withdrawn from trial treatment. Outcome assessments will be sought for all surviving participants, irrespective of compliance and domiciliary status and we estimate that 2-year assessments should be available on at least 80% of surviving participants (i.e. approximately 390) which would provide 90% power at $p<0.05$ to detect a 0.35 SD effect size reduction in the primary outcome measures, the decline in sMMSE and BADLS for minocycline compared

to placebo. The 12 month placebo decline on the BADLS seen in the AD2000 trial was about 10 points (SD 5.0), and thus a 0.35 SD effect size is equivalent to 1.8 BADLS points, or a reduction of about 20% in the rate of decline over 2 years, which is considered to be the minimum clinically important difference. Statistical power will be enhanced by use of repeated measures regression analyses including data at all time-points thus minimising the impact of participant attrition. With 130 patients allocated 400mg minocycline and 130 patients allocated 200mg minocycline assessed at 2 years, we will have 80% power at $p<0.05$ to detect a 0.35 SD effect size treatment effect of 400mg compared to 200mg (equivalent to about 3 points less decline with 400mg than placebo and 1 point less decline with 200mg than placebo).

7.2 Statistical Analysis

The hypothesis that minocycline reduces the rate of cognitive and functional decline, as measured on the sMMSE and BADLS, will be tested by comparing rates of decline over the two year study period. Primary analyses will use repeated measures regression techniques adjusted for baseline scores and the four minimisation factors. Participants will be analysed in the groups to which they are randomised irrespective of treatment compliance including data at all time points for all properly randomised patients, ie those who receive at least one dose of blinded trial medication, i.e. applying the principle of intention to treat as far as is practically possible, given any missing data. Patients who withdraw before receiving their treatment can be safely excluded as treatment allocation is double-blinded so cannot influence decision to withdraw and they have not, effectively, been randomised. For each outcome, the difference in rate of decline between minocycline (any dose) and placebo will be estimated. Similarly, the rates of decline in patients allocated 400mg and 200mg of minocycline will be compared. Time on trial medication will be compared between treatment arms using the log-rank test for equality of survivor functions. The incidence of serious adverse events will be compared between groups using Poisson regression. To explore the impact of missing data and treatment discontinuation, sensitivity analyses will be conducted. No adjustment for multiple significance testing will be made in the two primary analyses of the sMMSE and BADLS outcome measures. For secondary outcome measures, statistical significance will be defined as $p<0.01$ to compensate for multiple comparisons. Exploratory subgroup analyses of the influence of randomisation stratification variables on treatment efficacy will be undertaken and interpreted with appropriate caution. Full details of the statistical analysis will be set out in the MADE Statistical Analysis Plan.

7.3 Data Management

Paper case report forms (CRF) will be used and stored in a locked filing cabinet. The principal investigator for each site will act as custodian for the data and copies of the CRFs will be posted or faxed to the CTSU. Oxford CTSU will be responsible for the database and the resolution of data queries.

8) ORGANISATION

King's College London (KCL) and South London and Maudsley NHS Foundation Trust will act as co-sponsors. An independent TSC and DMEC will oversee the trial. The trial will adhere to the sponsor's SOP of trial management.

8.1 Local Principal Investigator

The local Principal Investigator (PI) is responsible for ensuring local compliance with GCP and research governance requirements. This includes obtaining management approval for MADE, ensuring that all members of the clinical team are familiar with the protocol and trial procedures, in particular serious adverse event reporting, maintaining the Local Study Site File with copies of trial materials, approval documents, consent forms and any other required documents as provided by the MADE Study office who will assist with gaining regulatory approval.

The PI will need to liaise with all who refer patients to the centre to encourage them to consider suitable patients for MADE. Recruitment into MADE should be facilitated by developing strong local links with non-teaching centres via DeNDRoN. Procedures will need to be developed to ensure assessment and discussion of individual patients' suitability for MADE at team meetings, providing eligible patients with MADE information sheets, arranging appointments to discuss taking part in the study, obtaining consent and randomisation, and delivering allocated drug packs to patients. Any member of the clinical team can obtain consent and randomise patients although it is obviously essential that teams liaise closely to agree who randomises and which patients are suitable for MADE.

The PI should ensure that medical and nursing staff involved in the assessment and treatment of patients with mild AD, are well informed about the study. This involves distributing the MADE materials to all relevant staff, displaying the wall-chart where it is likely to be read, and distributing the MADE newsletters. A regularly updated PowerPoint presentation will be provided to centres so that they can be shown from time to time, especially to new staff.

8.2 Local Study Coordinator

It is suggested that each Centre should designate one person as Local Study Coordinator (LSC). This role might suit a higher trainee in old age psychiatry or, if available, a research nurse. The LSC would be responsible for ensuring that all eligible patients are considered for MADE, that patients are provided with MADE information sheets and have an opportunity to discuss the study as required, obtaining consent, randomisation, obtaining drug packs from the pharmacy when patients are randomised, giving these to the patient with treatment instructions, and ensuring follow-up assessments are undertaken as scheduled in the protocol. The MADE LSC will also ensure that MADE trial forms, and questionnaires are completed and treatments are administered as scheduled (unless some contraindication develops). Again, this person would be sent updates and newsletters, would be invited to MADE progress meetings and appropriately credited in study reports.

8.3 Trial Steering Committee

The TSC is responsible for the independent supervision of the scientific and ethical conduct of the trial on behalf of the Trial Sponsor and the funding body, the Medical Research Council's EME programme. The TSC will review data, blinded to study treatment, on progress of the trial including recruitment, protocol adherence, serious adverse events, trial

publications and will determine the future progress of the trial in light of regular reports from the Trial Management Group (TMG) and DMEC. The TSC has the power to prematurely close the trial. The TSC will meet six-monthly or more often if the chair determines a reason for doing so. In addition to the independent voting members (listed inside front cover), the TSC will include the MADE CI, TM and Statistician, and representatives from the funding body and Sponsor. The TSC will report to the sponsors and the EME.

8.4 Data Monitoring and Ethics Committee

The independent DMEC (members listed inside front cover) is responsible for monitoring the unblinded accumulating data from the trial including: protocol adherence, SAEs and side effects of treatment as well as the difference between the trial treatments on the primary and secondary outcome measures. Based on the unblinded interim analyses, the DMEC can recommend protocol modifications to the TSC, including premature closure of the trial. The DMEC will agree their structure, organisation and stopping rules in a DMEC Charter (DAMOCLES Study Group, 2005) at their first meeting. The DMEC will first convene prior to trial initiation and will then define frequency of subsequent meetings (at least annually). The CI (or their representative) and the TM will be in attendance for the open session of the DMEC meeting. The trial statistician will be in attendance for the open session and to present and answer any questions on the interim analyses in the closed session. There will then be a session of just the independent members to agree any actions needed and the content of the DMEC report to the TSC. The DMEC will provide a recommendation to the TSC concerning the continuation of the study.

8.5 Ethics & Regulatory Approvals

The trial will be conducted in compliance with the principles of the Declaration of Helsinki (1996), the principles of GCP and in accordance with all applicable regulatory requirements including but not limited to the Research Governance Framework and the Medicines for Human Use (Clinical Trial) Regulations 2004, as amended in 2006 and any subsequent amendments.

This protocol and related documents have been reviewed and approved by:

(1) Essex Research Ethics Committee.

(2) The Medicines and Healthcare products Regulatory Agency (MHRA) for Clinical Trial Authorisation.

The integrated form for both site-specific assessment (SSA) and R&D approval at all participating NHS sites will also be approved prior to recruitment at each site. Annual progress and safety reports and a final report at conclusion of the trial will be submitted to the KHP-CTO (on behalf of the sponsor), the MREC and the MHRA within the timelines defined in the Regulations.

8.6 Quality Assurance

Recruitment to MADE and the conduct of trial assessments will be overseen by senior NHS clinicians who are experienced in the assessment and rating of psychopathology. All Investigators and staff employed on the grant will be trained in GCP, use of the assessment tools and safety monitoring and other trial procedures.

The Trial Manager will maintain a Trial Master File containing the essential trial documents in accordance with GCP and the EU Clinical Trial Directive. In addition, each site will be provided with an Investigator Site File and a Pharmacy File, which will contain the essential trial documents. The trial will be carried out in accordance with this protocol and other

MADE SOPs. Trial specific functions will be conducted in accordance with these and will ensure that the procedures within the trial are carried out in a standardised way in each centre.

Monitoring of this trial to ensure compliance with the protocol and GCP will be managed and overseen by the KHP-CTO Quality Team, in accordance with their SOPs, on behalf of the Sponsor. Each site will take part in a site initiation, to ensure appropriate staff training, resources, IMP management and essential documents are in place. During the course of the trial the study files will be reviewed for appropriate documentation of patient consent and participation in the trial, and a sample of data will be verified against patient notes in accordance with the risk assessment and monitoring plan for the trial. The Investigator(s) and the institutions will provide direct access to source data and other documents (e.g. patients' case sheets, etc.) to permit trial-related monitoring, audits, REC review, and regulatory inspections (where appropriate). At the end of the trial, each site will be formally closed down once trial activity at the site has ceased.

The CI will act as custodian for the trial data. All trial data will be stored on a password-protected computer and archived in line with the Medicines for Human Use (Clinical Trials) Amended Regulations 2006 as defined in the KHP-CTO Archiving SOP.

8.7 Publication Policy

The results of the study will be reported and disseminated at international conferences and in peer-reviewed scientific journals. A meeting of the TSC and MADE collaborators will be held after the end of the study to allow discussion of the main results prior to publication. The success of MADE depends entirely on the wholehearted collaboration of a large number of doctors, nurses and others. For this reason, chief credit for the main results will be given not to the committees or central organisers but to all those who have significantly contributed to the study. All grant holders and members of trial committees together with anyone who during the course of the study enters five or more patients into the study, and research workers at these centres who have been involved with the trial for more than 12 months, would have authorship rights as part of the MADE Trialists Group. Presentations or publications pertaining to the MADE trial must not be made without the prior agreement of the Trial Management Group.

8.8 Financial Aspects

Funding to conduct the MADE trial is provided by the NHS Efficacy and Mechanism Evaluation Programme (EME 11/47/01).

The duration of the grant is from 1st April 2013 until 31st March 2018. The grant will be administered by KCL and sub-contracts will be drawn up for the Study Office at CTSU, University of Oxford and for other sites.

8.9 Insurance/Indemnity

Kings College London indemnity applies for trial procedures and involvement.

9) REFERENCES

AD2000 Collaborative Group (2004). Long-term donepezil treatment in 565 patients with Alzheimer's disease (AD2000): randomised double blind trial. Lancet, 363, 2105-2115.

Aisen PS, Gauthier S, Ferris SH, et al (2011) Tramiprosate in mild-to-moderate Alzheimer's disease – arandomised double-blind placebo controlled multicentre study (the Alphase Study). Archives of Medical Science 7 102-111.

Bonelli RM, Hodl AK, Hofman P, Kapfhammer HP (2004) Neuroprotection in Huntington's disease: a 2-year study on minocycline. International Clinical Psychopharmacology 19 337-342.

Bucks RS, Ashworth DL, Wilcock GK, Siegfried K (1996) Assessment of activities of daily living in dementia: development of the Bristol Activities of Daily Living Scale. Age and Ageing 25 113-120.

Carty ML, Wixey JA, Colditz PB, Buller KM (2008) Post-insult minocycline treatmentattenuates hypoxia-ischaemia-induced neuroinflammation and white matter injury in the neonatal rat: a comparison of two different does regimens. International Journal of Developmental Neuroscience 26 477-485.

Casha S, Zygun D, McGowan D, et al (2009) Neuroprotection with minocycline after spinal cord injury: results of a double blind, randomized, controlled pilot study. Neurosurgery 65 410- 411.

Coi Y, Kim HS, Shin KY, et al (2007) Minocycline attenuates neuronal cell death andimproves cognitive impairment in Alzheimer's disease models. Neuropsychopharmacology 32, 2392-2404.

Cuello AC, Ferretti MT, Leon WC, et al (2010) Early stage inflammation and experimental therapy in transgenic models of the Alzheimer-like amyloid pathology. Neurodegenerative Disorders 7 96-98.

Diquet E, Fernagut PO, Wei X, et al (2004). Deleterious effects of minocycline in animal models of Parkinson's disease and Huntington's disease. European Journal of Neuroscience 19, 3266-3276.

Du Y, Ma Z, Lin S, et al (2001) Minocycline prevents nigrostriatal dopaminergic neurodegeneration in the MPTP model of Parkinson's disease. Proceedings of the National Academy of Sciences USA 98 14669-14674.

Familian A, Boschuizen RS, Eikelenboom P, Veerhuis R (2006) Inhibitory effect of minocycline on amyloid beta fibril formation and human microglial activation. Glia 53 233-240.

Familian A, Eikelenboom P, Veerhuis R (2007) Minocycline does not affect amyloid beta phagocytosis by human microglial cells. Neuroscience Letters 416 87-91.

Feldman H et al (2001). A 24-week randomized double blind study of donepezil in moderate to severe Alzheimer's disease. Neurology, 57, 613-620.

Folstein MF et al (1975). Mini-Mental state: a practical method for grading the cognitive state of patients for the clinician. Journal of Psychiatric Research, 12, 189-98.

Green RC, Schneider LS, Amato DA, Beelen AP, Wilcock G, Swabb EA, Zavitz KH (2009) Effect of Tarenflurbil on cognitive decline and activities of daily living in patients with mild Alzheimer's disease. A randomised controlled trial. JAMA 302 2557-2564.

Gordon PH, Moore DH, Miller RG, et al for Western ALS Study Group (2007) Efficacy of minocycline in patients with amyotrophic lateral sclerosis: a phase III randomised trial. Lancet Neurology 6 1045-1053.

Goulden V (1996) Safety of long-term high dose minocycline in the treatment of acne. British Journal of Dermatology 134 693-695.

Howard RJ et al (2007). Donepezil for the treatment of agitation in Alzheimer's disease. N Eng J Med 357, 1382-1392.

Howard R, Phillips P, Johnson T, et al (2010) Determining the minimum clinically important differences for outcomes in the DOMINO trial. International Journal of Geriatric Psychiatry 26, 812-817.

Howard RJ et al 2012. Donepezil and memantine for moderate-to-severe Alzheimer's disease. N Engl J Med. 366: 893-903.

Hunter CL, Quintero EM, Gilstrap L, Bhat NR, Grandhelm AC (2004) Minocycline protects basal forebrain cholinergic neurons from mu p75-saporin immunotoxic lesioning. European Journal of Neuroscience 19 3305-3316.

Huntington Study Group (2004) Minocycline safety and tolerability in Huntington disease.Neurology 63 547-549.
Lampl Y, Boaz M, Gilad R, et al (2007) Minocycline treatment in acute stroke:an open-label,evaluator-blinded study. Neurology 69 1404-1410.
Lechpammer M, Manning SM, Samonte F, et al (2008) Minocycline treatment following hypoxic/ischaemic injury attenuates white matter injury in a rodent model of periventricular leucomalacia. Neuropathology and Applied Neurobiology 34 379-393.
Liu Z, Fan Y, Won SL, et al (2007) Chronic treatment with minocycline preserves adult new neurons and reduces functional impairment after focal cerebral ischaemia. Stroke 38 146-152.
McKhann GM, Knopman DS, Chertkow H, et al (2011). The diagnosis of dementia due to Alzheimer's disease: recommendations from the National Institute on Aging-Alzheimer's Association workgroups on diagnostic guidelines for Alzheimer's disease. Alzheimers Dement 7 263-9.
Metz LM, Li D, Traboulsee A, et al for GA/minocycline study investigators (2009). Glatiramer acetate in combination with minocycline in patients with relapsing-remitting multiple sclerosis: results of a Canadian, multicenter, double-blind, placebo-controlled trial. Multiple Sclerosis 15 1183-1194.
Molloy DW et al (1991). Reliability of the standardized Mini-Mental State Examination compared with the traditional Mini-Mental State Examination. American Journal of Psychiatry, 148, 102-105.
Molloy DW, Standish TI (1997) A guide to the Standardised Mini-Mental State Examination. International Psychogeriatrics 9 Supplement 1 87-94.
NINDS NET-PD Investigators (2008) A pilot clinical trial of creatine and minocycline in early Parkinson disease: 18-month results. Clinical Neuropharmacology 31 141-150.
Noble W, Garwood CJ, Stephenson J, Kinsey AM, Hangar DP, Anderton BH (2009) Minocycline reduces the development of abnormal tau species in models of Alzheimer's disease. FASEB Journal 23 739-750.
Parachikova A, Visilevko V, Cribbs DH, Laferla FM, Green KN (2010) Reductions inamyloid-beta-derived neuroinflammation with minocycline restore cognition but do not significantly affect tau hyperphosphorylation. Journal of Alzheimer's Disease 21 527-542.
Plane JM, Shen Y, Pleasure DE, Deng W (2010) Prospects for minocycline neuroprotection. Archives of Neurology 67 1442-1448.
Radad K, Moldzio R, Rausch WD (2010) Minocycline prevents dopaminergic neurons against long-term rotenone toxicity. Canadian Journal of Neurological Science 37 81-85.
Ryu JK, McLarnon JG (2006) Minocycline or iNOS inhibition block 3-nitrotyrosine increases and blood-brain barrier leakiness in amyloid beta-peptide-injected rat hippocampus.Experimental Neurology 198 552-557.
Seabrook TJ, Jiang L, Maier M, Lemere CA (2006) Minocycline affects microglia activation, Abeta deposition, and behaviour in APP-tg mice. Glia 53 776-782.
Tombaugh TN and McIntyre NJ (1992). The Mini-Mental State Examination: a comprehensive review. Journal of the American Geriatric Society, 40, 922-935.
Tsuji M, Wilson MA, Lange MS, Johnson MV (2004) Minocycline worsens hypoxic ischaemic brain injury in a neonatal mouse model. Experimental Neurology 189 58-65.
Yang L, Sugama S, Chirichigno JW, et al (2003) Minocycline enhances MPTP toxicity to dopaminaregic neurons. Journal of Neuroscience Research 74 278-285.
Yrjanheikki J, Tikka T, Keinanen R, Goldsteins G, Chan PH, Koistinaho J (1999) A tetracycline derivative, minocycline, reduces inflammation and protects against focal cerebral ischaemia with a wide therapeutic window. Proceedings of National Academy of Sciences USA 96 13469-13500.
Zhu S, Stavrovoskaya IG, Drozda M, et al (2002) Minocycline inhibits cytochrome c release and delays progression of amyotrophic lateral sclerosis in mice. Nature 417 74-78.

MADE
Statistical Analysis Plan

Version Number: 1.0, 16th April 2018
Prepared by: Rosie Bradley and Richard Gray

1. Introduction

This document gives a detailed statistical analyses plan for the MADE trial, and should be read in conjunction with the current protocol.

2. Background

Alzheimer's disease (AD) is a major public health issue with approximately 700,000 people in the UK suffering from dementia of whom some 400,000 have Alzheimer's disease. With the population aging, the incidence of Alzheimer's disease is projected to increase with an estimated one million affected by 2020, at a cost of £20 billion a year. The imperative to discover and develop treatments that can stop or at least delay disease progression is clear. None of the drug treatments licensed for AD have been shown to affect progression of the illness and, despite a better understanding of the pathogenesis of AD, clinical trials of potentially disease modifying treatments so far undertaken have had disappointing results.
There is a substantial body of evidence to indicate that minocycline may be neuroprotective in neurodegenerative diseases such as AD. Although the primary neuroprotective target of minocycline in the central nervous system is not known, the principal effects of minocycline

include: inhibition of microglial activation, attenuation of apoptosis and suppression of the production of reactive oxygen species. Minocycline is arguably the most promising off patent candidate for AD modification that is not currently in trials and it is cheap and well tolerated.

3. Study Design

MADE is a multi-centre, randomised, controlled trial in very mild AD, which primarily aims to determine whether minocycline is superior to placebo in affecting the disease course, over a 2-year period, as measured by reduced rate of decline in:

I. Cognition (Standardised Mini-Mental State Examination (sMMSE))
II. Function (Bristol Activities of Daily Living Score (BADLS))

Data will be collected on the sMMSE at Screening, 6, 12, 18 and 24 months, and for the BADLS at Baseline, 6, 12, 18 and 24 months.

The secondary objectives of MADE are:

I. To compare the safety and tolerability of minocycline at doses of 400mg/day and 200mg/day
II. To determine whether a dose of 400mg/day offers superior neuroprotection to 200mg/day
III. To investigate associated risks and side-effects and serious adverse events
IV. To estimate the magnitude of any statistically significant positive treatment effects on cognitive and functional decline and thereby inform the design and powering of future phase III trial of definitive clinical effectiveness within the NHS

3.1 Treatment allocations

MADE is a pragmatic, phase II, 3-arm randomised, double-blind, multicentre study, with a semi-factorial design. Participants will be allocated to one of three arms:

Arm 1 – Minocycline 400mg/day
Arm 2 – Minocycline 200mg/day
Arm 3 - Placebo

3.2 Outcome measures

The first primary outcome measure is the standardised Mini-Mental State Examination (sMMSE), a widely used clinician-rated instrument for assessing cognitive ability. Scores range from 0 to 30 with higher scores indicating better cognitive function. There are 12 questions; if an answer is incorrect, incomplete or the participant is unable to answer the question, the questions receives a score of 0. The total score is the sum of the scores from each question. If the entire questionnaire has not been completed, the score at that visit will be set to missing.

The second primary outcome measure is the Bristol Activities of Daily Living Scale (BADLS), which is used to assess functional ability (activities of daily living). The questionnaire consists of 20 questions with four possible answers scored 0, 1, 2 or 3 or 'not applicable' which is scored 0. The score is the sum of these scores from each question ranging from 0 to 60 with higher scores indicating greater impairment. As the questionnaires includes the answer of 'not applicable' for every question, if the answer to any questions is missing, then the score is set to missing.

3.3 Sample size considerations

MADE aims to randomise at least 560 participants in a semi-factorial design (2x1) between minocycline (400mg), minocycline (200mg), and placebo minocycline. Even in mild AD, patient attrition is inevitable in a long term treatment trial and we estimate by 2 years, about 15% of patients will have died, a further 5% will be in institutionalisation care and about 40% will have withdrawn from trial treatment. Outcome assessment will be sought for all surviving patients, irrespective of compliance and domiciliary status, so we estimate that 2-year assessments will be available on at least 80% of surviving participants (i.e. approximately 390) which would provide 90% power at $p<0.05$ to detect a 0.35 SD effect size reduction in the primary outcome measures, the decline in sMMSE and BADLS for minocycline compared to placebo. With 130 patients allocated 400mg minocycline and 130 patients allocated 200mg minocycline assessed at 2 years, we will have 80% power at $p<0.05$ to detect a 0.35 SD effect size treatment effect of 400mg compared to 200mg.

4 General considerations

P-values:
Unless otherwise specified, estimates of minocycline efficacy will be presented with 95% confidence intervals. P-values will be from two-sided tests. As change in sMMSE is likely to

be correlated with change in BADLS, no corrections for multiple testing will be made in the co-primary analyses, though borderline significant p-values will be interpreted appropriately cautiously. For the secondary outcome measures, statistical significance will be defined as $p<0.01$ to compensate for multiple comparisons.

Protocol violations:
Primary analyses will be by intention to treat (ITT), including all patients who had started taking trial treatment. Patients who withdrew before receiving their treatment can be safely excluded as treatment allocation is double-blinded so cannot influence the decision to withdraw. Patients who start treatment will be analysed in the treatment arm to which they were randomised, and all patients shall be included whether or not they adhered to the treatment. This is to avoid any potential bias in the analysis. Secondary analysis such as 'per protocol' (only those patients who adhered with the treatment allocation) may be carried out but as a sensitivity analysis only.

Missing data:
The co-primary endpoints will be analysed using repeated measures analyses, including all available data, which minimises the impact of missing data. Since BADLS assessments are not valid for patients in institutional care, sensitivity analyses will be undertaken to explore the impact of assigning appropriately low scores for those with missing BADLS because they are in institutional care.

Late data:
The protocol allows for a 14-day leeway in the assessment dates. For late assessments, the actual date of the assessment will be used in repeated measures analyses, rather than assuming that the assessment was at the scheduled time point.

Timing of interim analyses:
Interim analyses of efficacy and safety will be undertaken at least annually as part of the DMEC reviews of accruing data. No formal stopping rules based on interim analyses will be applied. The DMEC will inform the Trial Steering Committee (TSC) if, in their view, information from unblinded analyses of interim data and other evidence from relevant studies provide proof beyond reasonable doubt that one or more of the treatments under investigation is either clearly indicated or contraindicated, either for all participants or for a particular subgroup of trial participants. This decision may in part be based on statistical considerations, but appropriate criteria for proof beyond reasonable doubt cannot be specified precisely, and will not be based on formal statistical stopping rules. Generally, a difference of at least three standard deviations in an interim analysis of a major endpoint is needed to justify halting, or modifying a study prematurely.

Timing of first main analyses for dissemination:
This will be undertaken once all patients have completed two years' follow up.

Unblinding of randomised treatments:
Any unblinding of randomised treatments will be recorded giving randomised group, centre, date and circumstances of unblinding.

5 Proposed Analyses

5.1 Co-primary endpoints:

The primary analyses of the effect of minocycline on the rate of decline of sMMSE and BADLS will use repeated measures regression methods, adjusted for baseline scores. This analysis uses all available visit data which will maximise statistical power to detect any differences between treatments. Time will be modelled as a continuous variable. Sensitivity analyses will also be undertaken including the minimisation factors (duration of symptoms, baseline sMMSE score and age) as covariates in the repeated measures model. A time by treatment interaction parameter will be included to examine if there is any changing treatment effect over time. Treatment effects are presented with 95% confidence interval and associated p-values in each case. For these analyses a p-value of 0.05 (5% level) will be used to indicate statistical significance

For both outcomes, the difference in the rate of decline between minocycline (any dose) and placebo will be estimated. Similarly, the rates of decline in patients allocated 400mg and 200mg of minocycline will be compared.

5.2 Secondary outcome measures:

(i) Time on trial medication will be compared between treatment arms using the log-rank test for equality of survivor functions

(ii) The incidence of adverse events and of serious adverse events will be compared between groups using Poisson regression

(iii) Time to entry into institutional care or death will be compared in log-rank analyses

The number of participants stopping trial medication over the 2 year period will be reported. Kaplan-Meier curves will be presented to show the time on trial medication split between the randomised groups. Statistical significance will be determined by log-rank test. Any participants who die during the trial will be censored at the date of death. Reasons for discontinuing from trial medication will be tabulated by randomised group.

All patients randomised in the trial (ie taking at least one dose of study treatment) are included in this safety analysis. Any serious adverse event (SAE) occurring whilst a participant is continuing in the study is included. All SAEs will be listed with details of event and randomised group. A comparison of serious adverse events between the randomised groups will be reported.

5.3 Sensitivity analyses:

To evaluate the effect of discontinuing study medication the primary analysis will be repeated on a sub-group of participants who adhere to their allocated treatment arm.

5.4 Planned subgroup analyses:

Subgroup analysis will be limited to the variables pre-specified in the protocol as minimisation variables used to balance randomisation:

- Duration of symptoms prior to randomisation (<6 months, ≥6 months)
- Baseline sMMSE score (24-26, 27-30)
- Age (<65, 65-74, ≥75 years)

These exploratory subgroup analyses will be undertaken, appropriately cautiously, to investigate any influence of the prognostic factors. These subgroup analyses will use standard test for significance of the parameter as an interaction in the repeated measures model.

Any deviations from this plan will be described in the final report.

This Statistical Analysis Plan has been read and approved by:

	**Name**	**Signed**	**Date**
**Senior Study Statistician:**	Richard Gray	Richard Gray	26/4/18
**Chief Investigator:**	Rob Howard		
**DMC chair (on behalf of the DMC)**	Peter Crome		
