# Supplementary material for: Minocycline at 2 Different Dosages vs Placebo for Patients With Mild Alzheimer Disease: A Randomized Clinical Trial
Source: JAMA Neurol. 2019 Nov 18;77(2):164–74. doi: 10.1001/jamaneurol.2019.3762 (PMC6865324; doi:10.1001/jamaneurol.2019.3762)
Supplement: Supplement 2. — eTable 1. Follow-up Rates for sMMSE and BADLS by Treatment Allocation and Follow-up Period eTable 2. Reasons for Failed Screens Into MADE Trial eTable 3. Causes of Death eTable 4. Baseline Characteristics of Those Who Stopped MADE Treatment eTable 5A. Line by Line Listings of Serious Adverse Events (SAEs) Categorised eTable 5B. Serious Adverse Events Categorised by Treatment Allocation and Whether on Treatment (IMP) eFigure 1. Flow Chart: Follow-up Completeness Over Time eFigure 2. Change in sMMSE From Baseline to Month 24 (A) Using Imputation Method (1) and (B) Using Imputation Method (2) to Estimate Scores for Patients With No Follow-up Past Baseline eFigure 3. Change in BADLS From Baseline to Month 24 (A) Using Imputation Method (1) and (B) Using Imputation Method (2) to Estimate Scores for Patients With No Follow-up Past Baseline eFigure 4. Subgroup Analyses of Change in sMMSE Over 24 Months for Minocycline (Any Dose) Versus Placebo by Baseline Characteristics eFigure 5. Probability of (A) Survival, (B) Remaining Community Resident and (C) Being Alive and Community-Resident by Treatment Allocation: Kaplan-Meier Survival Plots eFigure 6. Average Decline of sMMSE Split by Baseline sMMSE Score of 24-26 or 27-30 [file jamaneurol-77-164-s002.pdf]

Supplementary Online Content

Howard R, Zubko O, Bradley R, et al; Minocycline in Alzheimer Disease Efficacy (MADE) Trialist Group. Minocycline at 2 different dosages vs placebo for patients with mild alzheimer disease: a randomized clinical trial. *JAMA Neurol.* Published online November 18, 2019. doi:10.1001/jamaneurol.2019.3762

eTable 1. Follow-up Rates for sMMSE and BADLS by Treatment Allocation and Follow-up Period
eTable 2. Reasons for Failed Screens Into MADE Trial
eTable 3. Causes of Death
eTable 4. Baseline Characteristics of Those Who Stopped MADE Treatment
eTable 5A. Line by Line Listings of Serious Adverse Events (SAEs) Categorised
eTable 5B. Serious Adverse Events Categorised by Treatment Allocation and Whether on Treatment (IMP)
eFigure 1. Flow Chart: Follow-up Completeness Over Time
eFigure 2. Change in sMMSE From Baseline to Month 24 (A) Using Imputation Method (1) and (B) Using Imputation Method (2) to Estimate Scores for Patients With No Follow-up Past Baseline
eFigure 3. Change in BADLS From Baseline to Month 24 (A) Using Imputation Method (1) and (B) Using Imputation Method (2) to Estimate Scores for Patients With No Follow-up Past Baseline
eFigure 4. Subgroup Analyses of Change in sMMSE Over 24 Months for Minocycline (Any Dose) Versus Placebo by Baseline Characteristics
eFigure 5. Probability of (A) Survival, (B) Remaining Community Resident and (C) Being Alive and Community-Resident by Treatment Allocation: Kaplan-Meier Survival Plots
eFigure 6. Average Decline of sMMSE Split by Baseline sMMSE Score of 24-26 or 27-30

This supplementary material has been provided by the authors to give readers additional information about their work.

© 2019 Howard R et al. *JAMA Neurology*.

eTable 1. Follow-up rates for sMMSE and BADLS by treatment allocation and follow-up period

		sMMSE			BADLS		
		Received	Expected*	%	Received	Expected**	%
**Screening**	**400mg**	183	184	**99.5**	183	184	**99.5**
	**200mg**	181	181	**100**	181	181	**100**
	**Placebo**	178	179	**99.4**	177	178	**99.4**
	**Total**	**542**	**544**	**99.6**	**541**	**543**	**99.6**
**6 Month**	**400mg**	159	184	**86**	159	184	**86**
	**200mg**	172	181	**95**	172	181	**95**
	**Placebo**	167	179	**93**	164	176	**93**
	**Total**	**498**	**544**	**92**	**495**	**541**	**91**
**12 Month**	**400mg**	139	181	**77**	140	180	**78**
	**200mg**	158	180	**88**	157	178	**88**
	**Placebo**	156	176	**89**	155	171	**91**
	**Total**	453	537	**84**	452	529	**85**
**18 Month**	**400mg**	127	179	**71**	128	178	**72**
	**200mg**	146	177	**82**	146	169	**86**
	**Placebo**	147	172	**85**	148	167	**89**
	**Total**	**420**	**528**	**80**	**422**	**514**	**82**
**24 Month**	**400mg**	119	174	**68**	118	170	**69**
	**200mg**	144	176	**82**	142	167	**85**
	**Placebo**	140	167	**84**	137	154	**89**
	**Total**	**403**	**517**	**78**	**397**	**491**	**81**

***** Expected numbers of sMMSE assessments exclude those who withdrew prior to starting treatment – i.e., those not effectively randomised – and those who died prior to the assessment

** Expected numbers of BADLS* assessments also exclude those who were admitted to care

© 2019 Howard R et al. *JAMA Neurology*.

eTable 2. Reasons for failed screens into MADE trial

Reason for failed screen	Number of participants
**Ineligible**	
In another trial	7
Dementia diagnosis retracted due to lack of progression	7
No carer/study partner	8
Failed sMMSE	66
Lacks capacity/understanding	12
Allergies to antibiotic	2
eGFR too low	29
Blood test results ineligible	9
Physically too impaired to participate	8
Other conditions (e.g. cancer, mental health)	15
Patient in care	1
Not eligible for other reason	1
**Subtotal ineligible**	**165**
**Patient/ carer decision**	
Don't want any more medication	23
Patient doesn't want to take part – no reason given	60
Family reluctant	3
Don't want extra pressure/burden	4
Patient believes underlying physical problem	1
Feels dementia is not severe enough yet	2
Concerned about side effects	4
Still working, too busy	1
Study too long	1
**Subtotal Patient/ carer decision**	**99**
**Other**	
Moving out of area	1
Clinician refused to allow patient approach	1
Noncompliant with meds	1
Unable to contact again	10
**Subtotal other**	**13**
**Unknown**	**55**
**Total number of failed screens**	**332**
**Total screened**	**886**
**Total recruited**	**554**

© 2019 Howard R et al. *JAMA Neurology*.

eFigure 1. Flow chart: follow up completeness over time

Colour coding to show assessments split by treatment: red is 400mg, blue is 200mg and green is placebo.

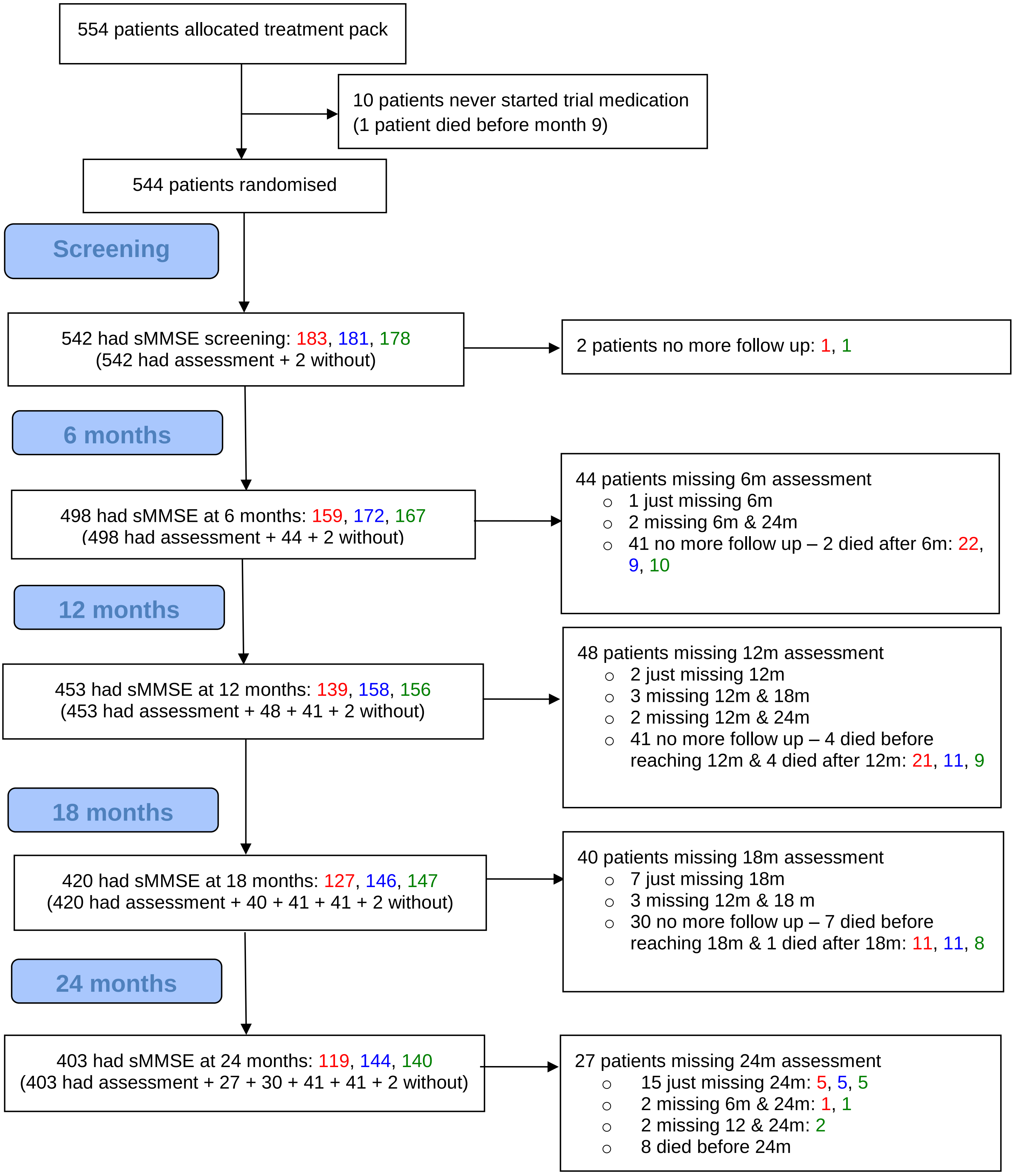

© 2019 Howard R et al. *JAMA Neurology*.

eTable 3. Causes of death

Treatment	Cause of death	Weeks until death	Stopped treatment ≥28 days previously?
**Infection**			
Placebo	Infection	64	Yes, 17 weeks
Placebo	Pneumonia	36	No
Placebo	Pneumonia and pulmonary oedema	28	Yes, 23 weeks
Placebo	Pneumonia	66	No
Placebo	Chest infection	83	No
200mg	Pneumonia	56	No
400mg	Pneumonia	86	Yes, 2 weeks
**Neuropsychiatric**			
Placebo	Dementia	95	No
Placebo	Alzheimer's/Lewy Body Dementia	92	Yes, 87 weeks
400mg	Progression of Alzheimer's	58	Yes, 7 weeks
**Cardiovascular**			
Placebo	Myocardial infarction	102	No
Placebo	Myocardial infarction	72	No
Placebo	Heart attack	64	No
200mg	Cardiac event	50	No
200mg	Heart attack	58	Yes, 51 weeks
400mg	Heart attack	37	No
400mg	Heart failure	100	Yes, 88 weeks
400mg	Heart attack	91	No
**Cerebrovascular**			
200mg	Unknown (stroke on 21/03/17)	103	Yes, 84 weeks
400mg	CVA	42	Yes, 3 weeks
400mg	Stroke	36	No
**Renal failure**			
Placebo	Chronic renal failure	32	Yes, 12 weeks
400mg	Lung and kidney failure	103	Yes, 1 week
**Other cause**			
Placebo	Complications after bowel surgery	89	Yes, 44 weeks
200mg	General health decline	56	Yes, 29 weeks
200mg	Large abdominal tumour causing kidney failure	28	Never started
400mg	COPD	57	Yes, 11 weeks
**Unknown**			
400mg	Unknown	77	Yes, 17 weeks

© 2019 Howard R et al. *JAMA Neurology*.

eFigure 2. Change in sMMSE from baseline to month 24 (A) using imputation method (1) and (B) using imputation method (2) to estimate scores for patients with no follow-up past baseline. Graph shows change in mean sMMSE scores with standard errors. Baseline scores* are set to zero. p-values are from tests for time-by-treatment interaction from repeated measures analysis. Results from ITT analysis N=554.

(2A) Imputation 1

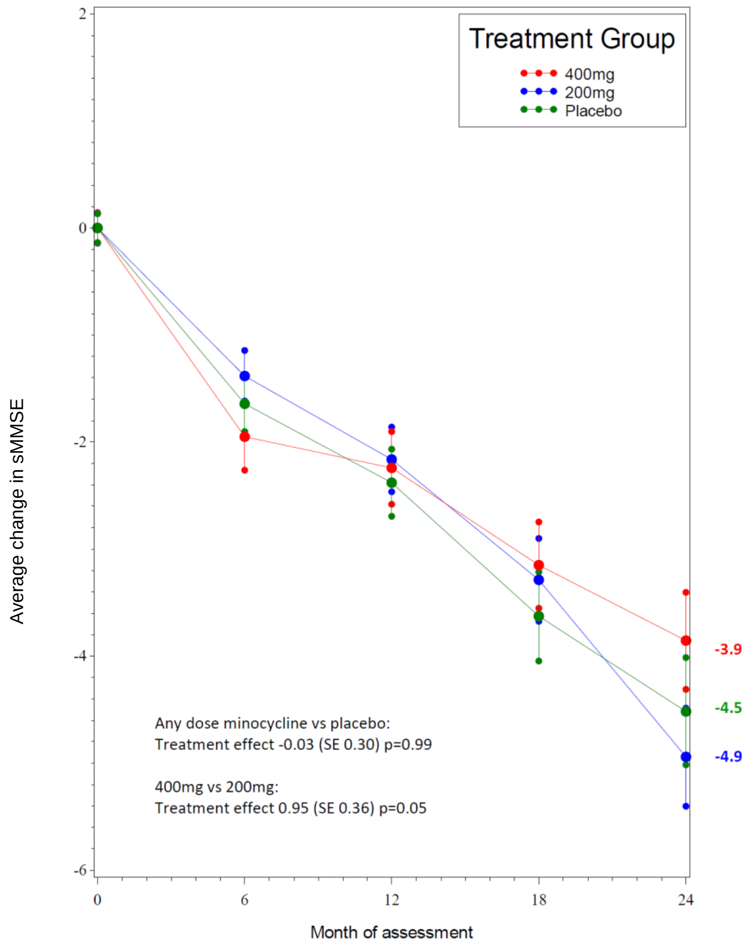

(2B) Imputation 2

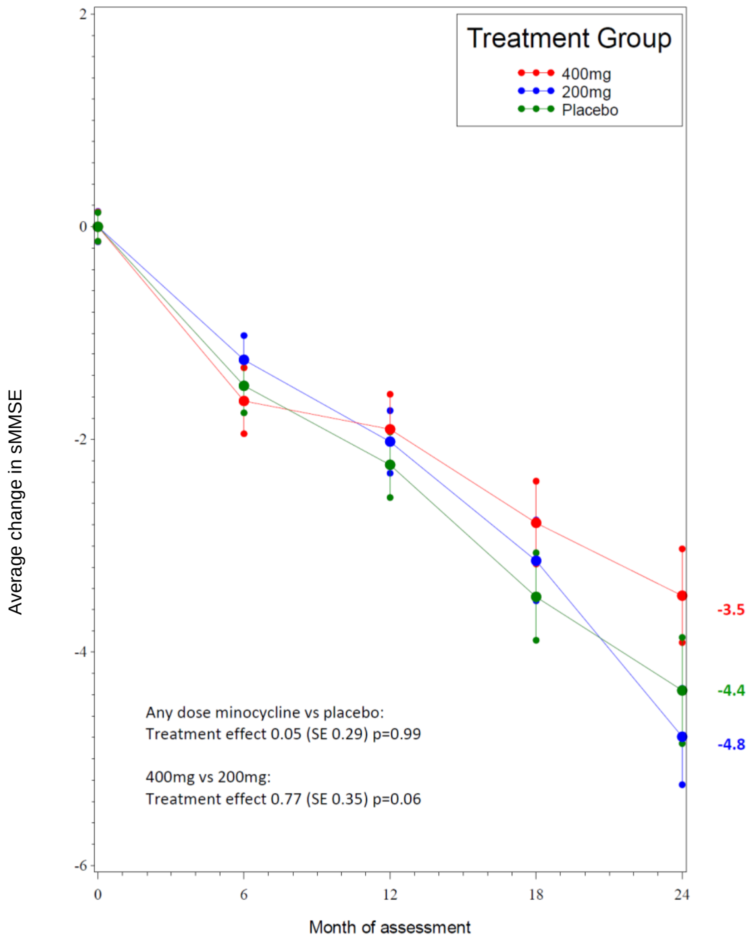

*Baseline scores: 400mg 26.3, 200mg 26.5, placebo 26.4

© 2019 Howard R et al. *JAMA Neurology*.

eFigure 3. Change in BADLS from baseline to month 24 (A) using imputation method (1) and (B) using imputation method (2) to estimate scores for patients with no follow-up past baseline. Graph shows change in mean BADLS scores with standard errors. Baseline scores* are set to zero. p-values are from tests for time-by-treatment interaction from repeated measures analysis. Results from ITT analysis N=554.

(2A) Imputation 1

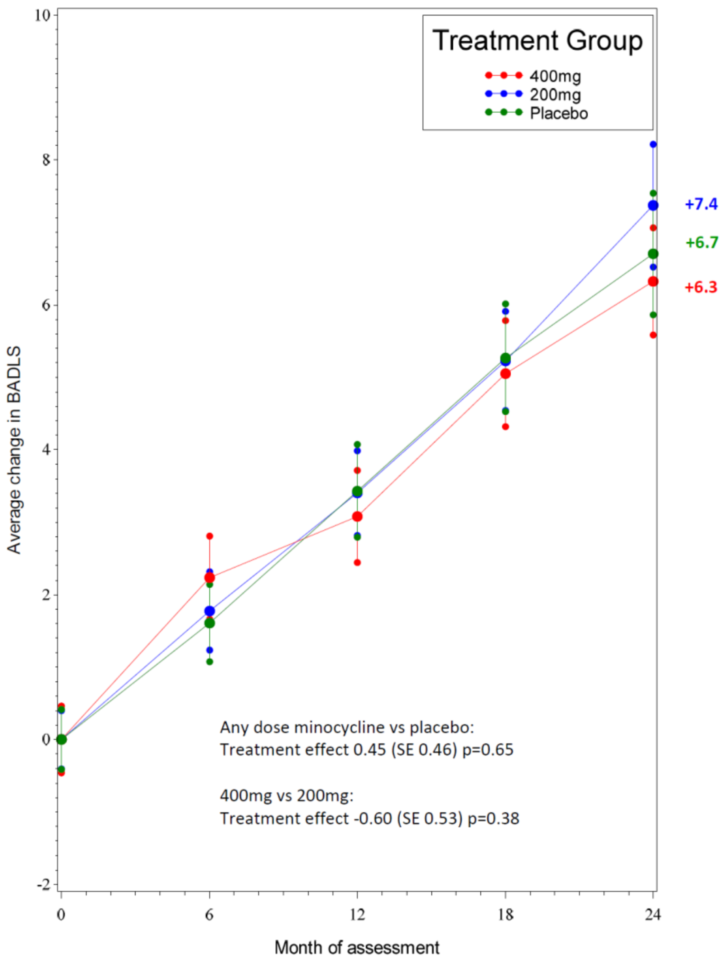

(2B) Imputation 2

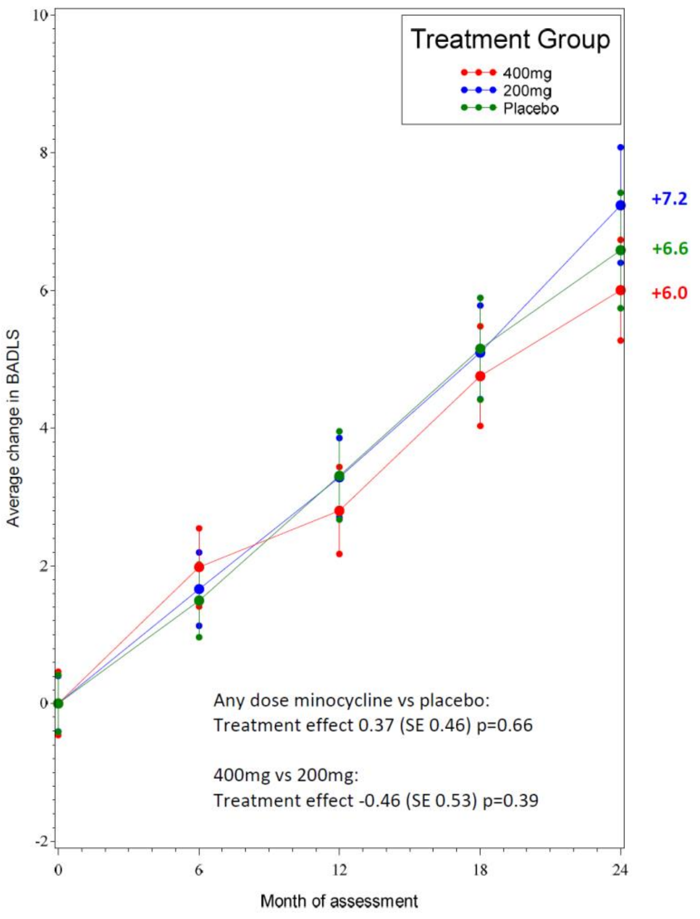

*Baseline scores: 400mg 5.6, 200mg 4.9, placebo 5.5

© 2019 Howard R et al. *JAMA Neurology*.

eFigure 4. Subgroup analyses of change in sMMSE over 24 months for minocycline (any dose) versus placebo by baseline characteristics: duration of symptoms, baseline sMMSE, age and gender. Results are derived from a repeated measures model, with p-values from tests for interaction between treatment and the selected subgroup.

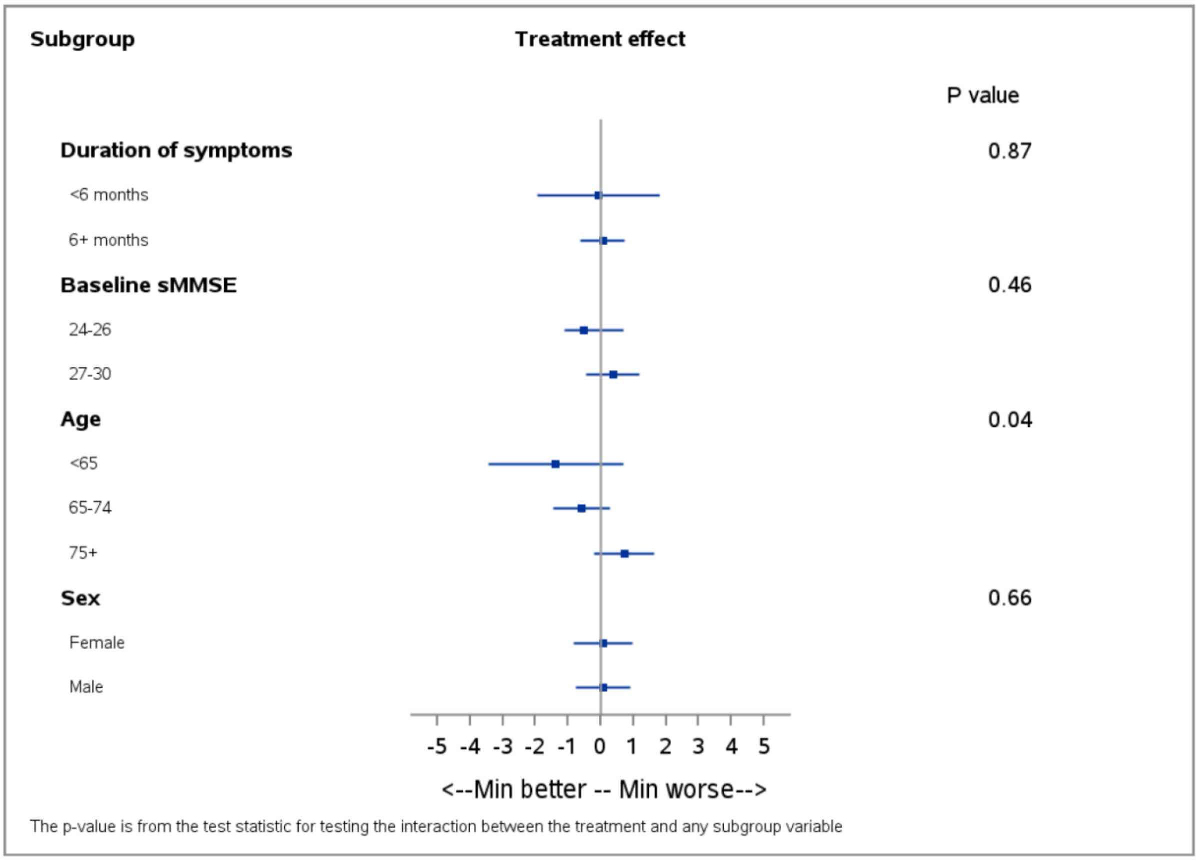

© 2019 Howard R et al. *JAMA Neurology*.

eFigure 5. Probability of A) Survival, (B) remaining community resident and (C) being alive and community-resident by treatment allocation: Kaplan-Meier survival plots

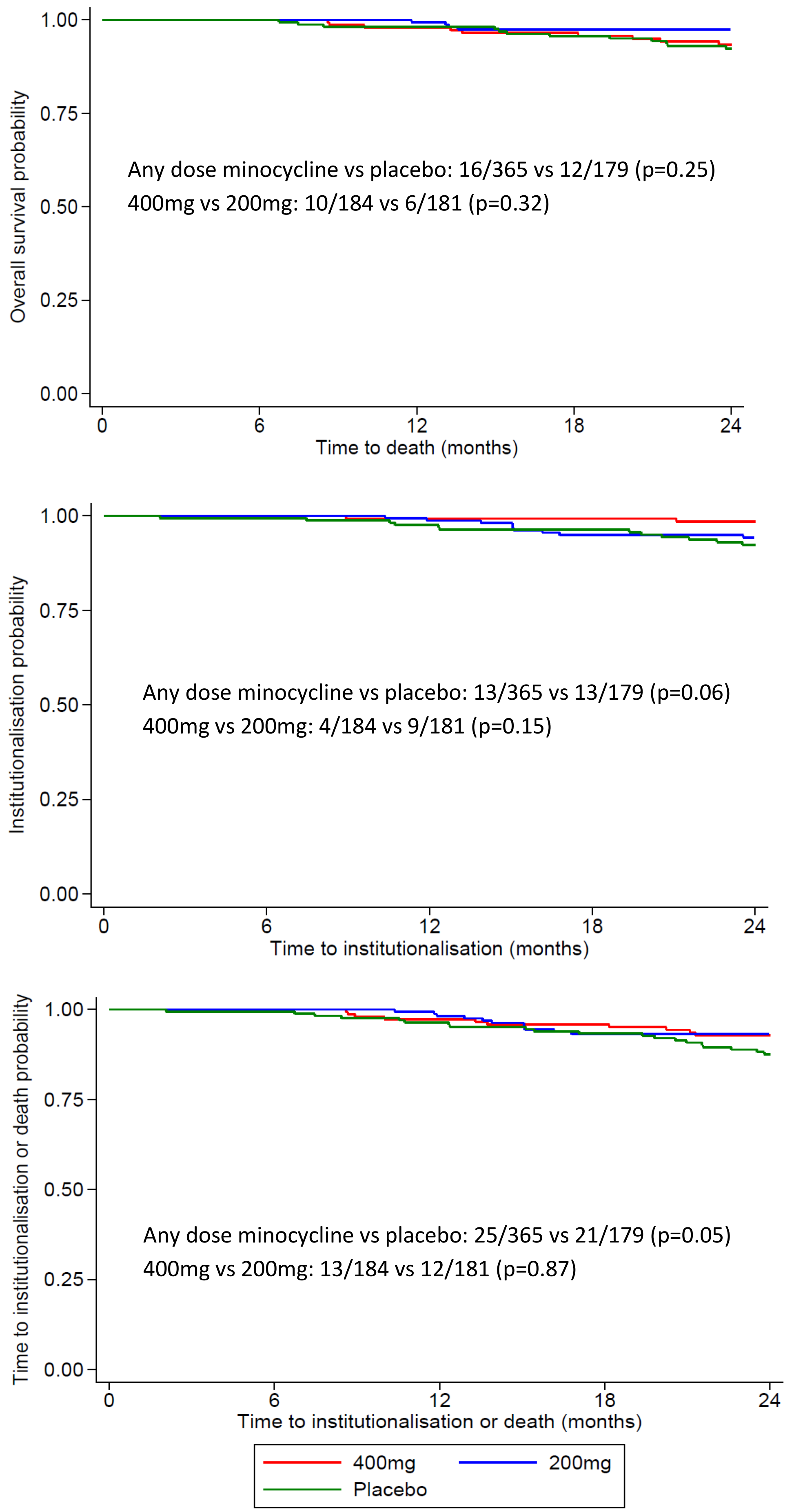

© 2019 Howard R et al. *JAMA Neurology*.

eFigure 6. Average decline of sMMSE split by baseline sMMSE score of 24-26 or 27-30*

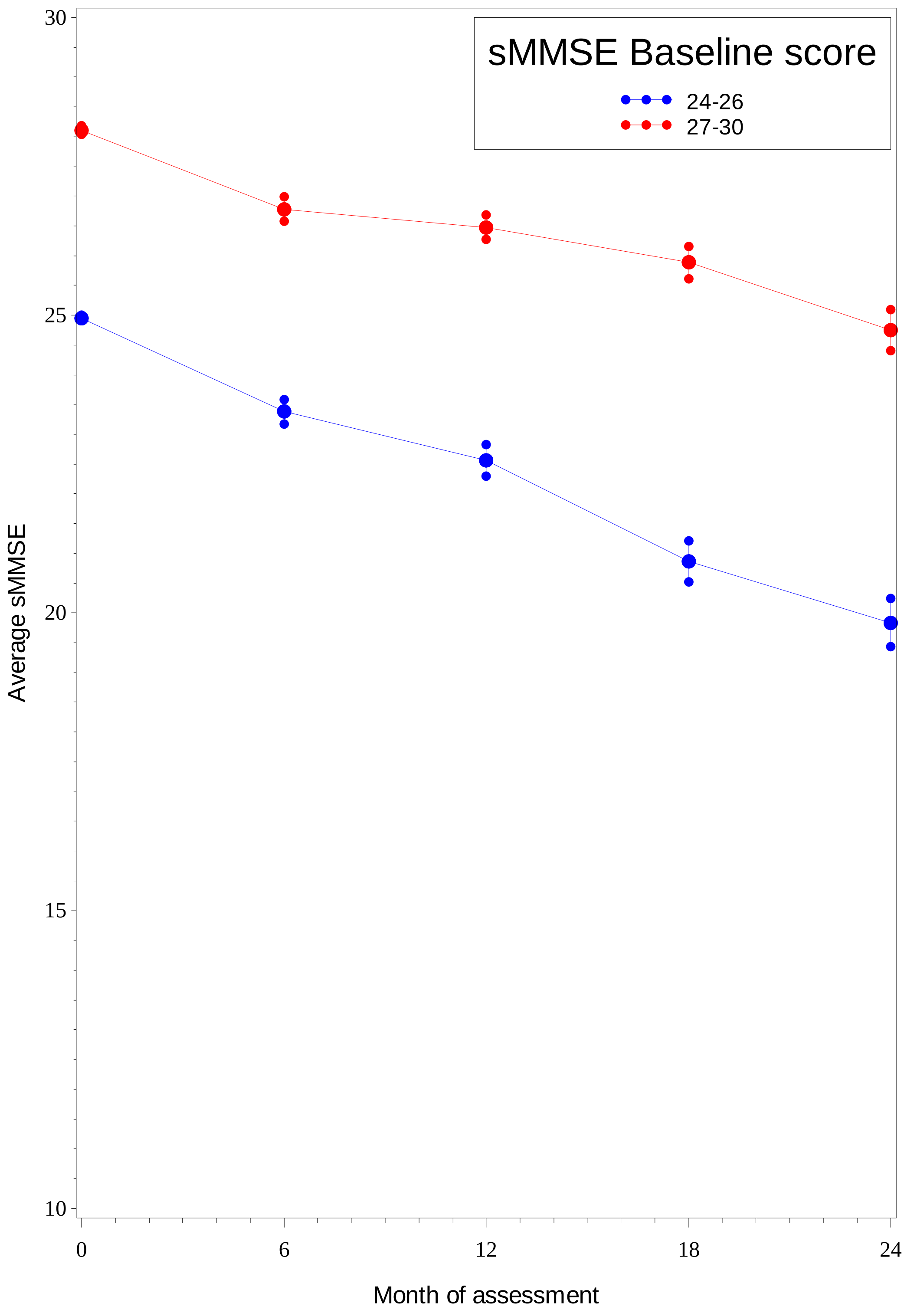

* 8 patients start with an sMMSE of 30 and have a 24 month sMMSE of 30

© 2019 Howard R et al. *JAMA Neurology*.

eTable 4. Baseline characteristics of those who stopped MADE treatment. Data are n (%).

Baseline characteristics		400mg (n=184)	200mg (n=181)	Placebo (n=179)	Total (n=544)
Age	<65 (n=65)	15 (23.1%)	7 (10.8%)	6 (9.2%)	28 (43.1%)
	65-74 (n=200)	45 (22.5%)	23 (11.5%)	19 (9.5%)	87 (43.5%)
	75+ (n=279)	68 (24.4%)	36 (12.9%)	35 (12.5%)	139 (49.8%)
Gender	Female (n=241)	56 (23.2%)	36 (14.9%)	27 (11.2%)	119 (49.4%)
	Male (n=303)	72 (23.8%)	30 (9.9%)	33 (10.9%)	135 (44.6%)
Duration of symptoms	<6 months (60)	14 (23.3%)	8 (13.3%)	7 (11.7%)	29 (48.3%)
	6+ months (n=484)	114 (23.6%)	58 (12.0%)	53 (11.0%)	225 (46.5%)

© 2019 Howard R et al. *JAMA Neurology*.

eTable 5A. Line by line listings of Serious Adverse Events (SAEs) categorised*

Reference Number	Event	On treatment?
**Gastrointestinal**		
SAE031	Gastroenteritis	Yes
SAE067	Constipation. Was taken to hospital, pt described medication.	Yes
SAE064	Gastroenteritis	Yes
SAE077	Sigmoid volvulus with faecal impaction	Stopped >28 days ago
SAE075	Diverticulitis and impacted bowel	Yes
SAE087	Deranged LFTs/stomach ulcer with gastrointestinal bleed	Yes (stopped same time)
SAE149	Abdominal distension pain	Stopped >28 days ago
SAE091	Constipation. Admitted to hospital with sickness/stomach pains.	Stopped <28 days ago
SAE095	Gradual internal bleeding of the stomach lining	Yes
SAE092	Diarrhoea and vomiting, and weight loss. Ambulance called and pt was admitted to hospital overnight. Hospital requested RNI scan.	Yes
SAE106	Under investigation – severe diarrhoea. Bowels going into spasms.	Yes
SAE112	Patient admitted to hospital with severe abdominal pains	Stopped >28 days ago
SAE120	Undiagnosed stomach pains. Investigations into possible stomach ulcer or reoccurrence of bowel cancer	Yes (stopped same time)
SAE138	Diverticulitis	Yes
SAE181	Gastroenteritis	Yes
SAE164	Appendicitis	Yes
SAE187	Death from complications after bowel surgery	Stopped >28 days ago
SAE209	Secondary, adhesion bowel obstruction – resulted in death	Stopped >28 days ago
SAE194	Bowel obstruction	Yes
SAE182	Cyst on small intestine updated diagnosis previously bowel obstruction	Yes
SAE228	Obstruction of the common bile duct	Yes
**Respiratory**		
SAE001	Pneumonia	Yes (stopped same time)
SAE002	Wheezing and shortness of breath	Yes (stopped same time)
SAE012	COPD	Yes
SAE020	Pneumonia	Yes
SAE018	Pneumonia – resulted in death	Yes (stopped same time)
SAE022	Suicide attempt and subsequent aspiration pneumonia and pulmonary oedema – resulted in death	Stopped >28 days ago
SAE025	Community acquired pneumonia	Yes (stopped same time)
SAE048	Sepsis secondary to community acquired pneumonia	Yes (stopped same time)
SAE043	Pneumonia	Yes
SAE047	Suspected pneumonia/further investigation	Yes
SAE079	Pneumonia and pleural effusion – resulted in death	Yes
SAE085	Pneumonia	Yes (stopped same time)
SAE116	Died – COPD	Stopped >28 days ago
SAE114	Pneumonia – died in hospital	Yes (stopped same time)
SAE117	Pneumonia preceded by declining neutrophil count which then rose before ceasing IMP	Yes
SAE136	Community acquired pneumonia	Yes
SAE122	Pneumonia	Yes
SAE156	Increased shortness of breath	Stopped <28 days ago
SAE163	Breathlessness & extreme thirst	Yes
SAE184	Pneumonia	Yes
SAE200	Chronic obstructive pulmonary disease	Yes
SAE226	Admitted to hospital after fall contracted pneumonia while in hospital	Yes
SAE266	Admitted to hospital after fall & contracted pneumonia while in hospital. Had fractured rib	Yes

© 2019 Howard R et al. *JAMA Neurology*.

SAE243	Pneumonia	Stopped >28 days ago
SAE270	Pulmonary fibrosis	Yes
SAE215	Pneumonia – resulted in death	Stopped >28 days ago
**Mechanical injury**		
SAE004	Fall resulting in skull fracture	Yes
SAE015	Fall and closed fracture of rib	Yes (stopped same time)
SAE021	Fall out of bed resulting in head and neck injury	Stopped <28 days ago
SAE034	Fall sustaining cuts, bruising and reduced mobility	Yes
SAE053	Unwitnessed fall out of bed	Yes
SAE068	Fractures pubic rami after a fall	Yes
SAE046	Fracture of right femur from a fall	Yes (stopped same time)
SAE054	Fractured wrist from a fall	Yes
SAE080	Patient fell and fractured pelvis, related to increased dizziness, less steady on feet since starting MADE treatment	Stopped <28 days ago
SAE063	Possible bruised, cracked or broken ribs following a fall due to tripping	Yes
SAE073	Shoulder surgery following fall and dislocation	Yes
SAE065	Fracture right humerus from a fall	Yes
SAE093	Cerebral concussion and cut to head from a fall	Yes
SAE124	Fractured right neck of femur	Yes (stopped same time)
SAE155	Road traffic accident – patient hit by car	Stopped >28 days ago
SAE119	Fall and admission to hospital overnight	Yes
SAE139	Fractured left femur and underwent left hemiarthroplasty	Yes
SAE175	Fractured ribs from a fall	Yes
SAE117	Broken left hip	Yes
SAE196	Fracture of left neck of femur	Yes
SAE199	Knee replacement operation	Yes
SAE222	Fall	Yes
SAE232	Fracture of metacarpal	Yes
SAE235	Facial injury from fall	Stopped >28 days ago
SAE237	Collapse & facial injury and nasal fracture	Yes
SAE254	Fractured hip	Yes
SAE255	Fall causing pubic ramus and wrist fracture	Yes
SAE258	Vertigo/dizziness – leading to head injury from a fall	Stopped >28 days ago
SAE256	Fracture to middle finger and left hand	Yes
SAE263	Mechanical fall and back pain	Stopped >28 days ago
**Endocrine and metabolic**		
SAE008	Diabetes mellitus management impairment – resolved	Yes
SAE007	New medical diagnosis of type 2 diabetes	Yes
SAE060	Low sodium levels	Yes
SAE061	Pituitary adenoma	Stopped >28 days ago
SAE056	Diabetes ketoacidosis	Stopped >28 days ago
SAE072	Hypoglycaemia	Stopped >28 days ago
SAE118	Hypoglycaemia	Yes
SAE132	Syndrome of inappropriate antidiuretic hormone (SIADH)	Yes
SAE135	Low potassium due to bowel preparation for CT bowel	Yes
SAE189	Admitted to hospital after feeling weak and faint. Diagnosed with low sodium levels	Yes
SAE190	Admitted to hospital following low sodium levels and generally feeling weak	Yes
SAE249	Inflammatory arthropathy – likely due to gout	Yes
**Cancer**		
SAE005	Reoccurrence of bladder cancer	Yes
SAE032	Tumour on kidney – right kidney/part of liver removed	Yes
SAE024	Colon cancer	Yes
SAE040	Colon cancer (open anterior resection surgery)	Yes
SAE066	Chronic lymphocytic leukaemia	Yes
SAE096	Diagnosis of myeloproliferative neoplasm JAK-1	Yes
SAE078	Working diagnosis – colon cancer. Pt feels well so pt/family don’t want further tests	Yes

© 2019 Howard R et al. *JAMA Neurology*.

SAE083	Bowel cancer – resulted in death	Yes
SAE011	Cancer of oesophagus	Stopped >28 days ago
SAE123	Suspected kidney cancer, diagnosis of left renal tumour	Stopped >28 days ago
SAE229	Chronic lymphoid leukaemia	Stopped >28 days ago
SAE102	Recent lung cancer diagnosis. Further investigation of cancer shows it to be terminal with secondary's in the liver	Yes
SAE109	Bowen's disease	Yes
SAE183	Myelodysplastic syndrome (MDS)	Yes
SAE147	Bowel cancer	Stopped >28 days ago
SAE130	Probable lung cancer, won't undergo treatment for cancer	Stopped >28 days ago
SAE142	Patient diagnosed with prostate cancer	Yes
SAE161	Prostate cancer with pelvic metastasis	Yes
SAE191	Colonic primary tumour, with extensive liver metastasis	Stopped <28 days ago
SAE193	Complex atypical hyperplasia	Yes (stopped same time)
SAE206	Lung tumour and secondary cancers	Yes
SAE212	Appearances consistent with lung malignancy. Given co-morbid condition for best supportive/palliative care	Yes
SAE217	Vulvar cancer	Yes
SAE238	Tonsillectomy due to cancer	Yes
SAE251	Prostate cancer	Stopped >28 days ago
SAE253	Basal cell carcinoma	Yes
**Haematological/thrombosis**		
SAE038	Low platelets	Yes (stopped same time)
SAE045	Blood transfusion for suspected bleed, following low haemoglobin	Yes
SAE110	DVT	Yes (stopped same time)
SAE129	Neutropenia	Yes
SAE145	Anaemia	Yes
SAE236	Blood clot	Yes
**Dermatological**		
SAE245	Minocycline type 2 pigmentation on face	Yes
**Neuropsychiatric**		
SAE009	Hospitalisation following seizures	Yes
SAE019	Psychosis secondary to dementia. Also mild UTI	Yes (stopped same time)
SAE029	Admitted to psychiatric unit following relapse in psychotic symptoms with agitation	Stopped >28 days ago
SAE128	Patient confused, lacked coordination and had been experiencing more falls for a few weeks	Yes
SAE160	Hospital admission with severe Alzheimer's dementia with significant behavioural disturbance	Stopped >28 days ago
SAE050	Admission to hospital due to loss of consciousness	Yes
SAE158	Death due to dementia	Yes
SAE016	Subdural haematoma	Yes (stopped same time)
SAE152	Alzheimer's disease/Lewy body disease – resulted in death	Stopped <28 days ago
SAE143	Stroke	Yes (stopped same time)
SAE105	Probable stroke. Also receiving treatment for chest infection	Yes (stopped same time)
SAE055	Minor stroke	Yes
SAE146	Seizure (known epilepsy), cracked bone in ankle	Yes
SAE086	CVA – resulted in death	Stopped >28 days ago
SAE170	Alzheimer's disease	Yes
SAE076	Alzheimer's disease	Yes
SAE203	Minor stroke non haemorrhagic	Yes
SAE069	Stroke	Yes

© 2019 Howard R et al. *JAMA Neurology*.

SAE059	Left intra-cranial bleed	Yes (stopped same time)
SAE154	Alzheimer's disease	Yes
SAE166	Funny turns followed by suspected TIA. Patient hospitalised	Stopped >28 days ago
SAE150	Stroke	Yes
SAE167	Bleeding on brain	Yes
SAE159	Mini stroke (TIA)	Yes
SAE169	Suspected stroke/seizure	Yes
SAE172	Stroke – resulted in death	Yes
SAE088	Small left frontal lobe cortical haemorrhage	Yes
SAE153	Subdural haemorrhage/blood clot	Yes
SAE121	Admitted following falls, is due to be discharged home with end of life/full time carers. MRI showed chronic subdural haematoma	Yes (stopped same time)
SAE134	Seizure, no diagnosis given	Stopped <28 days ago
SAE218	Delirium	Yes (stopped same time)
SAE219	Dementia in Alzheimer's disease	Yes
SAE198	Confusion, slurred speech, unsteady on feet. Admitted to hospital	Yes
SAE230	Patient in acute psychiatric ward on section 2	Yes
SAE231	Suspected stroke. Patient died	Stopped >28 days ago
SAE239	Possible TIA	Yes
SAE247	Delirium	Yes
SAE252	Progression of Alzheimer's disease – resulted in death	Yes (stopped same time)
SAE265	CVA	Yes (stopped same time)
**Cardio-circulatory**		
SAE036	Suspected myocardial infarction	Yes
SAE030	Shortness of breath and suspected MI	Yes
SAE010	Cardiac abnormalities: Long QT on ECG and impaired left ventricular function	Yes
SAE062	Cardiac event – resulted in death	Yes (stopped same time)
SAE035	Myocardial infarction	Yes
SAE099	Syncope attributed to GTN spray overdose (accidental)	Yes
SAE090	Swollen ankles, shortness of breath, ambulance called. Admitted to hospital for 11 days. Diagnosis fluid on lungs and heart murmur	Yes
SAE104	Cardiogenic syncope	Yes
SAE094	Collapsed, thought to be due to low blood pressure	Yes
SAE098	Postural hypotension	Yes
SAE201	Out of hospital ventricular fibrillation arrest due to anterior myocardial infarction – resulted in death	Yes (stopped same time)
SAE211	Recurrent gradual onset syncope, junctional bradycardia on implantable loop recorder	Yes
SAE208	Postural hypotension	Yes
SAE039	Hospitalisation - low blood pressure/pulse rate	Yes
SAE157	Hypotension	Yes
SAE125	Chest pain	Yes
SAE049	Suspected heart attack resulting in death	Yes (stopped same time)
SAE137	Severe mitral valve regurgitation, which resolved upon rate-limiting control of AF and LV improvement in function	Yes
SAE144	Currently unknown – heart related problems	Yes
SAE151	Death from MI	Yes (stopped same time)
SAE168	Death – coronary atherosclerosis hypertension	Yes (stopped same time)
SAE171	Cardiac arrest	Yes
SAE207	Heart problems. Cardiac monitor had revealed that heart had stopped for short time	Yes
SAE202	Heart failure	Stopped <28 days ago
SAE205	Heart failure	Stopped <28 days ago
SAE176	Aortic stenosis	Yes
SAE103	Deterioration in cardiac failure plus syncopal episode lead to hospital admission	Yes (stopped same time)
SAE224	Heart failure – resulted in death	Stopped >28 days ago

© 2019 Howard R et al. *JAMA Neurology*.

SAE241	Heart attack – resulted in death	Stopped >28 days ago
SAE240	Labile blood pressure/hypertension	Yes
SAE246	Heart failure	Yes
SAE248	Heart attack – resulted in death	Yes (stopped same time)
SAE564	Syncope (due to likely bradycardia)	Stopped >28 days ago
SAE268	Atrial fibrillation	Stopped >28 days ago
**Renal**		
SAE057	Kidney stones	Yes
SAE058	Large abdominal tumour causing kidney failure – resulted in death	Never started
SAE084	Chronic renal failure – resulted in death	Stopped >28 days ago
SAE101	No evidence of bladder cancer. Pt was having tests from a bladder biopsy.	Yes
SAE113	Radical left nephrectomy laparoscopy	Stopped >28 days ago
SAE204	Acute kidney injury	Yes
SAE267	Lung and kidney failure – resulted in death	Stopped >28 days ago
**Infection**		
SAE014	Hospitalisation – chest infection	Yes
SAE017	Hospitalisation – delirium due to dehydration & urine infection	Yes
SAE044	Probable UTI, symptoms of confusion, weakness, low mobility	Yes
SAE082	Infection following a foreign body in arm	Yes
SAE051	Chest infection	Yes (stopped same time)
SAE052	Urinary tract infection	Yes (stopped same time)
SAE081	Catheter associated UTI	Yes (stopped same time)
SAE070	Chest infection	Yes
SAE097	Infection – resulted in death	Stopped >28 days ago
SAE100	Pruritic rash in the context or urosepsis	Yes
SAE107	Urosepsis	Yes
SAE115	Sepsis, possibly related to gall bladder problems	Stopped >28 days ago
SAE131	Progressive decline post chest infection	Stopped >28 days ago
SAE127	Urinary infection	Stopped <28 days ago
SAE141	Admitted with lower respiratory infection	Yes (stopped same time)
SAE165	Patient admitted to hospital with very sore throat later diagnosed as thrush	Yes
SAE173	Shortness of breath and chest infection	Yes
SAE174	Shortness of breath and chest infection	Yes
SAE180	Taken to hospital with very low blood pressure and infection	Yes
SAE188	Sepsis	Stopped >28 days ago
SAE195	11/11/16 Wife reports participant has chest infection – resulted in death	Yes (stopped same time)
SAE197	Urinary tract infection	Yes
SAE133	Updated from discharge summary: Cellulitis	Stopped >28 days ago
SAE233	UTI	Stopped >28 days ago
SAE234	UTI	Stopped >28 days ago
SAE210	Urinary tract infection, confusion	Yes
SAE216	Oesophageal candidiasis	Yes
SAE213	Admitted with lower respiratory tract infection	Stopped >28 days ago
SAE22	UTI	Yes
SAE223	UTI	Yes
**Other**		
SAE006	Hospitalisation – collapsed in street	Yes
SAE028	Suspected blood clot in legs. Swollen legs/painful. No blood clot found	Yes

© 2019 Howard R et al. *JAMA Neurology*.

SAE033	Suspected thrombosis – investigations complete no diagnosis of thrombosis. Symptoms of swollen legs have been associated with previously known water condition. Symptoms reduced after treatment.	Yes
SAE042	Participant drank white spirit in error	Yes
SAE071	Replacement of left knee	Stopped >28 days ago
SAE111	Sensitivity and tenderness around left nipple	Yes
SAE074	IMP overdose	Yes
SAE089	Patient collapsed following accidental overdose of ranitidine	Yes (stopped same time)
SAE108	General health decline– resulted in death. Pt was in respite care.	Stopped >28 days ago
SAE148	Osteoarthritis	Yes
SAE179	Jaw, back and neck pain – no diagnosis – cardiac problems ruled out	Stopped >28 days ago
SAE162	Prolonged hospital stay after planned hernia operation	Yes
SAE186	Admitted for elective abdominal hysterectomy and bilateral salpingo-oophorectomy	Yes
SAE178	No acute medical problem identified. Pt felt as if she had severe indigestion. Investigations into whether it was a mild heart attack in A&E (stayed overnight). No explanation found.	Stopped >28 days ago
SAE185	(TURP) Transurethral resection of the prostate	Yes
SAE214	Hip screw being removed	Yes
SAE225		Yes
SAE244	Post op – scrotal oedema	Yes
SAE242	Swollen legs	Yes
SAE250	Unknown – resulted in death	>28 days ago

* Participants can have more than one recorded SAE in each category

© 2019 Howard R et al. *JAMA Neurology*.

eTable 5B. Serious Adverse Events categorised by treatment allocation and whether on treatment (IMP)

	Counts of SAEs reported							
SAE class	400mg		200mg		Placebo		Total	
	On IMP	OFF IMP	On IMP	OFF IMP	On IMP	OFF IMP	**On IMP**	**OFF IMP**
Gastrointestinal	2	1	7	1	4	6	**13**	**8**
Respiratory	6	2	6	2	9	1	**21**	**5**
Mechanical injury	4	2	9	2	11	2	**24**	**6**
Endocrine and metabolic	1	1	1	0	7	2	**9**	**3**
Cancer	8	4	2	1	9	2	**19**	**7**
Haematological/thrombosis	3	0	1	0	2	0	**6**	**0**
Dermatological	0	0	1	0	0	0	**1**	**0**
Neuropsychiatric	9	1	8	5	15	1	**32**	**7**
Cardio-circulatory	10	4	8	1	10	1	**28**	**6**
Renal	1	2	1	1	1	1	**3**	**4**
Infection	5	5	0	1	16	3	**21**	**9**
Other	5	2	8	3	2	0	**15**	**5**
**Total**	**54**	**24**	**52**	**17**	**86**	**19**	**192**	**60**

© 2019 Howard R et al. *JAMA Neurology*.
