# Supplementary material for: Minocycline at 2 Different Dosages vs Placebo for Patients With Mild Alzheimer Disease: A Randomized Clinical Trial
Source: JAMA Neurol. 2019 Nov 18;77(2):164–74. doi: 10.1001/jamaneurol.2019.3762 (PMC6865324; doi:10.1001/jamaneurol.2019.3762)
Supplement: Supplement 3. — Data Sharing Statement [file jamaneurol-77-164-s003.pdf]

Data Sharing Statement

Howard. Minocycline at 2 Different Dosages vs Placebo for Patients With Mild Alzheimer Disease. *JAMA Neurol*. Published November 18, 2019. 10.1001/jamaneurol.2019.3762

Data
Data available: Yes
Data types: Deidentified participant data
How to access data: robert.howard@ucl.ac.uk
When available: With publication

Supporting Documents
Document types: None

Additional Information
Who can access the data: Researchers whose proposed use of the data has been approved.
Types of analyses: For meta-analyses or any analyses of the data approved by the Trial Steering Committee.
Mechanisms of data availability: With investigator support.
